# Recent Advances in Molybdenum Disulfide and Its Nanocomposites for Energy Applications: Challenges and Development

**DOI:** 10.3390/ma16124471

**Published:** 2023-06-19

**Authors:** Kamal Batcha Mohamed Ismail, Manoharan Arun Kumar, Shanmugam Mahalingam, Junghwan Kim, Raji Atchudan

**Affiliations:** 1 Department of Electrical, Electronics and Communication Engineering, School of Technology, Gandhi Institute of Technology and Management (GITAM), Bengaluru 561203, Karnataka, India; mohamedismailmk@gmail.com; 2 Department of Electronics and Communication Engineering, Agni College of Technology, Chennai 600130, Tamil Nadu, India; 3 Department of Materials System Engineering, Pukyong National University, Busan 48513, Republic of Korea; shanmugam.mr3@gmail.com (S.M.); junghwan.kim@pknu.ac.kr (J.K.); 4 School of Chemical Engineering, Yeungnam University, Gyeongsan 38541, Republic of Korea; 5 Department of Chemistry, Saveetha School of Engineering, Saveetha Institute of Medical and Technical Sciences, Chennai 602105, Tamil Nadu, India

**Keywords:** molybdenum disulfide, nanocomposites, energy storage, supercapacitors

## Abstract

Energy storage and conversion are critical components of modern energy systems, enabling the integration of renewable energy sources and the optimization of energy use. These technologies play a key role in reducing greenhouse gas emissions and promoting sustainable development. Supercapacitors play a vital role in the development of energy storage systems due to their high power density, long life cycles, high stability, low manufacturing cost, fast charging-discharging capability and eco-friendly. Molybdenum disulfide (MoS_2_) has emerged as a promising material for supercapacitor electrodes due to its high surface area, excellent electrical conductivity, and good stability. Its unique layered structure also allows for efficient ion transport and storage, making it a potential candidate for high-performance energy storage devices. Additionally, research efforts have focused on improving synthesis methods and developing novel device architectures to enhance the performance of MoS_2_-based devices. This review article on MoS_2_ and MoS_2_-based nanocomposites provides a comprehensive overview of the recent advancements in the synthesis, properties, and applications of MoS_2_ and its nanocomposites in the field of supercapacitors. This article also highlights the challenges and future directions in this rapidly growing field.

## 1. Introduction

The global energy requirement is continuously on the rise because of the burgeoning population, expanding urban areas, and the growth of industries. This has led to a greater need for renewable energy sources and energy-efficient technologies to meet the demand while reducing carbon emissions. Non-renewable resources such as coal, oil, and natural gas are categorized as fossil fuels that took millions of years to form from the decomposed remains of dead animals and plants. These fuels are major sources of energy for electricity generation, transportation, and industrial processes, but their use contributes to climate change and environmental degradation. Fossil fuels still dominate the global energy mix and account for the majority of energy production in the world [1,2,3]. In addition to being finite resources that will eventually run out, fossil fuels also contribute to air pollution and climate change through the release of greenhouse gases during combustion. These negative impacts have spurred a global shift towards renewable energy sources. The sustainable and environmental advantages of renewable energy sources like solar, wind, and hydropower are causing them to gain more popularity [4,5].

Renewable energy sources provide several advantages, including decreasing the amount of greenhouse gas emissions, enhancing the quality of air, and generating employment opportunities in the clean energy industry. Additionally, renewable energy is often more cost-effective and sustainable in the long term compared to traditional fossil fuels [6]. With the increasing shift towards renewable sources of energy, energy storage is gaining more importance. The intermittent nature of wind and solar power underlines the amplified requirement for energy storage to balance out the supply and demand of energy and ensure the reliable and consistent availability of power [7]. Insufficient energy storage is a major challenge for renewable energy sources, as they are dependent on weather conditions. Developing cost-effective and efficient energy storage technologies is crucial to overcoming this challenge and making renewable energy more reliable and accessible [8]. Energy storage devices are designed to accumulate extra energy during periods of low consumption and restore it during periods of high demand. This process helps maintain a balanced grid and prevent power outages. This technology is becoming increasingly important as more renewable energy sources are added to the grid, which can be intermittent and unpredictable [9].

There are three types of energy storage devices: mechanical, electrical, and chemical. Flywheels and compressed air energy storage fall under the category of mechanical storage systems, while batteries and supercapacitors are classified as electrical storage systems [10]. Both batteries and capacitors are energy storage devices, but their energy densities and discharge rates differ. Batteries have a higher energy density and slower discharge rate, while capacitors have a lower energy density and faster discharge rate [11]. Batteries store the charges electrochemically. This means that they use chemical reactions to produce and store electrical energy, which can then be used to power various devices and equipment. Additionally, different types of batteries have varying capacities and lifetimes, making them suitable for different applications [12]. Capacitors store charges electrostatically and are commonly used in electronic circuits to smooth out voltage fluctuations and filter out unwanted signals [13]. They come in various sizes and types, including ceramic [14], electrolytic [15], and tantalum capacitors [16].

**Figure 1:**
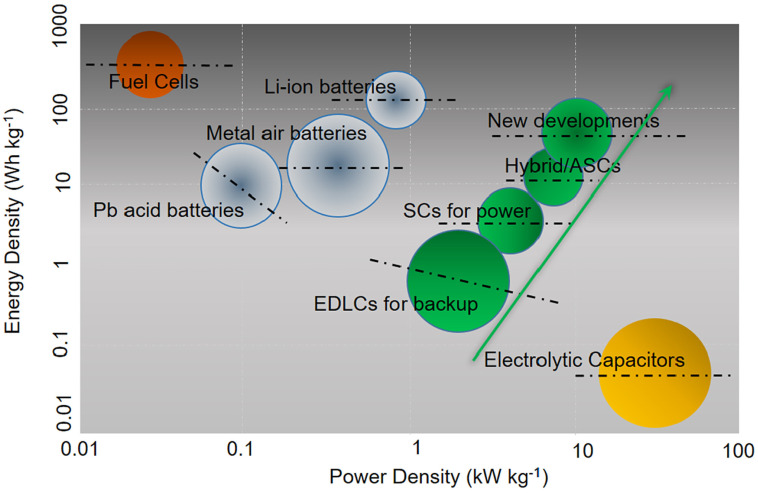
The Ragone plot displays the relationship between energy and power density of various energy storage devices. Adapted from [20]. Copyright 2020, Energy Reports.

Supercapacitors are high-capacity electrochemical devices that store and release energy quickly. They have the ability to store and deliver much higher energy densities than conventional capacitors due to their unique electrode configurations. Supercapacitors typically have larger capacitance values compared to traditional capacitors, and their charge/discharge cycles are much faster. There are three types of supercapacitors, which are categorized as electrostatic double-layer capacitors, pseudo-capacitors, and hybrid capacitors. Electrostatic double-layer capacitors are the most common type of supercapacitor. They operate by separating positive and negative charges over two layers of electrically conducting material separated by a thin separator. This separation creates a high-capacity electrical field, which stores charge. Pseudo-capacitors, on the other hand, work by storing charge on the surface of a conducting material through a process called faradaic charge transfer. This type of supercapacitor has a higher energy density than electrostatic double-layer capacitors but typically has a lower power density. Hybrid capacitors, as the name suggests, combine the features of both electrostatic double-layer capacitors and pseudo-capacitors. By doing so, they are able to offer not only high energy density but also high power density. This makes them ideal for high-performance applications which require both high energy and power density [17]. The use of supercapacitors has been on the rise recently as they possess the capacity to quickly store and distribute energy, making them a suitable option for electric vehicles and sustainable energy setups. As the world shifts towards more sustainable forms of energy, the demand for supercapacitors is expected to continue to grow [18]. Supercapacitors have several advantages over traditional batteries, including faster charging times, longer lifetimes, and higher power densities. They are also more environmentally friendly, as they do not contain toxic chemicals or heavy metals [19]. In the energy storage industry, a Ragone plot is frequently utilized to assess the viability of various technologies by illustrating the compromise between power density and energy density. It compares the appropriateness of different energy storage systems for different applications. This plot (Figure 1) is also employed to gauge the potential of new materials and technologies in the field of energy storage [20].

### Transition Metal Dichalcogenides (TMDs)

Transition metal dichalcogenides (TMDs) are a group of 2D materials that are made up of a transition metal atom like molybdenum or tungsten sandwiched between two layers of chalcogen atoms, such as sulfur or selenium. They display distinctive features in terms of their electrical, optical, and mechanical characteristics, which make them highly potential materials for applications in electronics [21], bio-applications [22], topological spintronics [23], optoelectronics [24], energy storage [25], and energy conversion devices [26]. Over the years, researchers have successfully synthesized stable TMDCs using a variety of methods like chemical vapor deposition (CVD), chemical exfoliation, and solvothermal synthesis. The utilization of these techniques permits the accurate manipulation of the size, morphology, and structure of the TMDs, ultimately influencing their characteristic traits [27]. TMDs like MoS_2_, WS_2_, MoSe_2_, and WSe_2_ have been extensively studied because of their extraordinary electronic, optical, and mechanical characteristics that are prominently displayed when their thickness is decreased to just a few layers [28]. TMDs have a layered crystal structure, with strong covalent bonds within the layers and weak van der Waals forces between the layers. This leads to a favorable relationship between surface area and volume, resulting in optimal mechanical flexibility. TMDs also have a direct bandgap, which makes them efficient light absorbers and emitters [29,30].

The edge locations exhibit a significant concentration of atoms that are unsaturated, thereby offering a greater number of active sites for chemical reactions. Additionally, the coordination of the edge atoms is different from that of the basal atoms, resulting in a different electronic environment. This causes the edge sites to have a different electrochemical potential, making them more active in redox reactions [31]. The fabrications of electrodes using TMDs are often optimized to incorporate the edge-exposed surface of these materials, as opposed to their basal planes. This strategy can enhance both their catalytic and electrochemical properties. The edge sites of TMDs possess higher reactivity and greater activity towards electrochemical reactions compared to the basal plane sites [32,33]. In recent years, there has been a noteworthy rise in the studies conducted on two-dimensional TMDs, particularly MoS_2_, with a focus on their electrochemical properties [34]. Molybdenum disulfide (MoS_2_) displays enormous potential in diverse electrochemical applications such as batteries [35], hydrogen evolution [36], supercapacitors [37], and sensors [38]. The unique atomic structure of MoS_2_, which consists of a layered structure of molybdenum and sulfur atoms, provides it with exceptional electro catalytic properties. MoS_2_-based electrochemical devices have shown remarkable performance in terms of rapid charge/discharge rates, high energy density, and extended life cycles. Researchers are also exploring the use of MoS_2_ in hybrid energy storage systems [39], ionic-liquid-based supercapacitors [40], and as a catalyst for water splitting [41] and carbon dioxide reduction [42].

**Figure 2:**
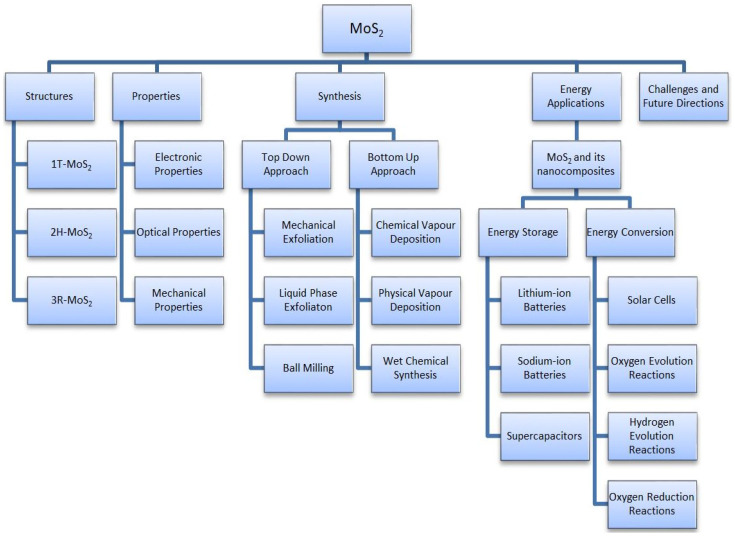
Schematic representation of overview of this review article.

This article provides a general overview of the various 2D TMDs, focusing on their significant structure of MoS_2_. The mechanical, electrical, and optical properties of MoS_2_ and their impact on various applications are discussed in detail. Additionally, the paper systematically outlines the latest advancements in the methods used for their preparation and highlights their primary advantages and disadvantages. The main focus of this article is to explore the applications of MoS_2_ and its nanocomposites in energy storage and conversion techniques. In this study, we extensively evaluate the electrochemistry and capabilities of wearable and flexible supercapacitors based on MoS_2_, which have not been previously discussed in other review articles. Finally, we discussed the real-time challenges and the research gaps to improve the performance of MoS_2_ and its nanocomposites for potential applications in industries. The schematic representation of the overview of this review article is shown in Figure 2.

## 2. Structures, Properties and Synthesis of MoS_2_

### 2.1. Structure of MoS_2_

**Figure 3:**
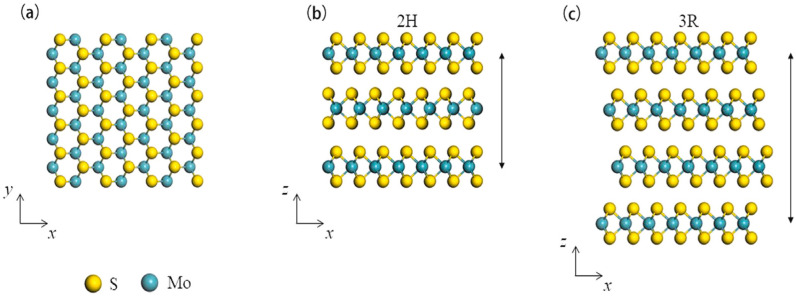
(**a**) Top view of monolayer MoS_2_ with stick and ball crystal structure, (**b**) Side view of multilayer MoS_2_ with 2H structure, (**c**) Side view of multilayer MoS_2_ with 3R structure. The arrow marks indicate the number of layers in a repeat unit. Adapted from [45]. Copyright 2019 Nano Energy.

MoS_2_ is a unique material that exhibits different properties and applications depending on its structure and dimensions. The structure is composed of layers that are arranged in a hexagonal lattice made up of molybdenum atoms, with two sulfur atoms in between each layer. MoS_2_ has three polymorphic crystalline structures, which are 1T-MoS_2_, 2H-MoS_2_ and 3R-MoS_2_. 1T-MoS_2_ is the most thermodynamically stable polymorph of MoS_2_ at high temperatures. The Mo atoms are arranged in trigonal prismatic coordination, with a distorted octahedron formed by the six closest sulfur atoms. Its crystal structure is characterized by the existence of one type of sulfur atom in the lattice [43]. 2H-MoS_2_ is the most common form of MoS_2_ in nature. It has a hexagonal crystal structure, and each Mo atom is surrounded by six sulfur atoms forming a trigonal prism arrangement. Its unit cell consists of two different sulfur sites in the lattice [44]. 3R-MoS_2_ polymorph has a rhombohedral crystal structure (Figure 3) and is found at low temperatures. It has a lattice parameter that lies between the 1T and 2H structure and three different sulfur sites in the lattice [45].

One of the most common and stable phases of MoS_2_ is known as the 2H structure. This structure has a hexagonal lattice, which has two layers per unit cell. The adjacent layers are stacked in an ABA pattern, in which the middle layer is shifted slightly in relation to the top and bottom layers. This arrangement allows for strong interlayer interactions between the sulfur atoms, resulting in a stable crystalline structure [46]. The sulfur atoms from different atomic planes occupy the same positions relative to each other. The Mo-S bond in MoS_2_ is predominantly covalent in nature, meaning that the electrons are shared between the two atoms. This results in strong bonding between the Mo and S atoms within each sheet, contributing to the strong mechanical properties and thermal stability of the material. On the other hand, the layers of S atoms are coupled by van der Waals interactions, which are weaker and result from the temporary dipole moments that arise when the electron cloud around the atoms is slightly displaced [47]. The layered structure of MoS_2_ turns it into a perfect material for various applications, and its morphology can be controlled by different synthesis methods. MoS_2_ can be synthesized in various shapes and morphologies, including planar [48,49,50], nanoflowers [51], nanowires [52], nanotubes [53], nanoplatelets [54,55] and vertically aligned nanosheets [56]. Overall, the different structures of MoS_2_ offer a wide range of possibilities for scientific research and technological innovation.

### 2.2. Properties of MoS_2_

The properties of MoS_2_ play a crucial role in determining its suitability for energy storage applications. MoS_2_ possesses exceptional attributes, including high electrochemical activity, fast ion diffusion, and outstanding electrical conductivity, which make it a highly favored choice as an electrode in batteries and supercapacitors. The characteristics of MoS_2_ have a considerable impact on the utilization of energy storage in various applications, mainly because of its vast surface area that allows for a substantial active area for electrochemical reactions. Thus, the electrodes made of MoS_2_ possess a high capacitance and can conveniently accumulate a significant amount of charge, a crucial aspect for the superior functioning of energy storage devices. Another important property of MoS_2_ is its high mechanical strength and stability, which enables it to withstand repeated cycles of charge and discharge with minimal degradation. Additionally, MoS_2_ has good thermal stability and is capable of operating at high temperatures without compromising its performance, making it suitable for use in harsh environments. In addition, MoS_2_ has an electronic band structure that gives it a high electron affinity. This property makes it possible to achieve high energy storage density and excellent charge retention, which are essential for the development of high-performance energy storage devices [57,58,59].

#### 2.2.1. Electronic Properties

The properties of multilayer MoS_2_ have been widely examined because of its impressive electronic and optical characteristics. Its band structure has been a focus of interest due to its potential use in optoelectronic devices. Studies have shown that multilayer MoS_2_ has an indirect band gap of 1.2 eV, indicating that the transfer of electrons from the valence to conduction bands necessitates a shift in momentum. However, the band gap increases as the number of layers in MoS_2_ decreases. MoS_2_ in a single-layer configuration, also known as monolayer MoS_2_, demonstrates a direct band gap measuring 1.8 eV. Moreover, the thickness-dependent band gap of MoS_2_ makes it possible to engineer its optical and electronic properties by controlling the number of layers and even stacking different materials on top of each other [60,61]. However, compared to silicon, which has a direct bandgap of 1.12 eV, MoS_2_ still falls short in terms of its ability to efficiently absorb and emit light in the visible spectrum [62]. The electronic characteristics of MoS_2_ can be significantly impacted by mechanical strain, which refers to the deformation caused by an applied force. Specifically, it can cause a change in the material’s band gap. For MoS_2_, this strain can shift its band gap from a direct one to an indirect one, thereby affecting its electronic properties in a significant way. Specifically, it can turn the material from a semiconductor into a metal, where there is no longer a band gap, and electrons can move freely across the material. This change in properties could have significant implications for the use of MoS_2_ in electronic devices, making it possible to tune its conductivity and optoelectronic properties through the application of strain. In addition to mechanical strain, other factors, such as electric fields and chemical doping, can also influence the electronic characteristics of MoS_2_ [63].

Monolayer MoS_2_ has become a noteworthy semiconductor material in recent times owing to its distinct characteristics. When this material is doped with chromium, copper, or scandium, it undergoes a transition to become an n-type semiconductor. On the other hand, doping with nickel or zinc causes monolayer MoS_2_ to become a p-type semiconductor. The ability to control the electrical properties of monolayer MoS_2_ through the process of doping opens up many potential applications in electronic devices. For example, n-type MoS_2_ can be used as a component in transistors, while p-type MoS_2_ can be used to create diodes [64]. The doping of Titanium in MoS_2_ can significantly alter its electronic properties, and the type of doping (interstitial or substitutional), as well as the doping concentration, play a crucial role in the resulting semiconductor behavior. Low levels of Ti doping lead to p-type behavior, while high levels result in n-type and, eventually, a ferromagnetic half-metal with spin polarization equal to one. These results may pave the way for the creation of advanced electronic gadgets that offer better performance and functionality [65].

#### 2.2.2. Optical Properties

MoS_2_ is a semiconducting material that exhibits interesting optical properties. One of its notable characteristics is its absorption coefficient—a measure of how much light is absorbed as it passes through the material. The absorption coefficient of MoS_2_ was influenced by various factors, such as the chemical composition of the material, its thickness, and the wavelength of light being used. In the case of MoS_2_, its absorption coefficient is relatively large for wavelengths ranging from 400 nm to 500 nm [66]. The bandgap of bulk MoS_2_ changes when it is reduced to a few layers. This bandgap change in MoS_2_ enables the tunability of its photoresponsivity, specific detectivity, and response time. The photoresponsivity of MoS_2_-based devices is enhanced by the reduction in thickness due to quantum confinement effects. This confinement effect increases the electronic density of states in the bandgap, leading to an increase in light absorption and an increase in photoresponsivity. In addition to photoresponsivity, the specific detectivity of MoS_2_-based devices is improved by the increased surface area when reduced to a few layers, which improves the signal-to-noise ratio. The response time of MoS_2_-based devices is improved due to the reduction in thickness, which allows for faster carrier transport and recombination. Furthermore, the high mobility of charge carriers in MoS_2_ also contributes to its fast response time. Overall, the potential for MoS_2_ to be used in optoelectronics is very high due to its ability to have a tunable bandgap. This material has promising applications in photodetectors, solar cells, light-emitting diodes, and even optical switches and modulators [67].

The electronic and structural properties of MoS_2_ can be effectively assessed using its photoluminescence (PL) spectra. Any alterations in the material’s band gap, doping, or crystal structure can greatly impact its PL activity, making it a valuable tool for characterization. In pure MoS_2_, the band gap is estimated to be around 1.8 eV, resulting in an emission of around 670 nm. However, MoS_2_ can be doped with various elements, such as nitrogen or sulfur, which can alter the band structure and shift the emission peak. For example, nitrogen-doped MoS_2_ exhibits a blue-shifted PL peak due to a reduced band gap [68]. Moreover, the PL activity of MoS_2_ can also be affected by its structure. MoS_2_ can exist in different crystal forms, such as 1T or 2H, which have different electronic properties and, therefore, different PL activities. According to reports, the PL intensity of 1T-MoS_2_ is stronger as compared to 2H-MoS_2_ [69]. The addition of an H_2_O_2_ solution has been found to be an effective approach for enhancing the photoluminescence properties of monolayer MoS_2_. This is because H_2_O_2_ acts as a strong oxidizer that oxidizes the MoS_2_ surface without altering its crystalline structure [70]. Amani et al. attained an important improvement in the quality of MoS_2_ by using a chemical treatment of an organic super-acid, resulting in a quantum yield (QY) of 95%. This means that almost all of the absorbed energy is emitted as light, indicating a highly efficient process. It was noticed that the lifespan of MoS_2_ carriers is nearly 10.8 nanoseconds, making it a suitable material for utilization in advanced solar cells and high-performance lasers [71]. MoS_2_ exhibits exceptional electrical and optical characteristics, which make it a highly desirable substance for optoelectronic applications. However, the presence of defects has been a major obstacle to the realization of its full potential.

#### 2.2.3. Mechanical Properties

The exclusive structure of Monolayer MoS_2_ contributes to its remarkable mechanical strength, which is similar to that of graphene, a two-dimensional material renowned for its exceptional strength. The Young’s modulus of a single layer of MoS_2_, which measures its stiffness and resistance to deformation, has been found to be 0.33 ± 0.07 TPa (terapascals). This is a remarkable value, particularly given that the Young’s modulus of steel, a material commonly used in construction, is only around 200 GPa (gigapascals). The lattice structure of MoS_2_ enables the material to withstand large amounts of stress without breaking or deforming [57]. One notable property of MoS_2_ is that it undergoes a transition from a bulk material to a two-dimensional (2D) material when its thickness is reduced to a single layer. This change is significant as the mechanical properties of MoS_2_ are affected: single-layered structures are more flexible than bulk materials. This is predominantly because of the interlayer interactions that exist in the bulk form of MoS_2_, resulting in a stiffer structure, whereas in the form of a single layer, the feeble van der Waals forces between layers enable increased flexibility [72].

One of the ways to alter the electronic characteristics of MoS_2_ is through mechanical strain. If mechanical stress is applied to MoS_2_, its band gap transforms from direct to indirect. This change in the band gap can result in new electronic and optical properties. Furthermore, when MoS_2_ is subjected to high strain values, it can undergo a structural deformation that can transform it from a semiconductor to a metal. This is because the deformation alters the Mo-S bonds and changes the electronic states of the material. Furthermore, the mechanical strain can also change the lattice symmetry, which influences the electronic properties of the material [73]. One of the significant differences between MoS_2_ monolayers and graphene is their thermal conductivity. MoS_2_ monolayers have a thermal conductivity of around 35 Wm^−1^ K^−1^, which is approximately 100 times lower than the thermal conductivity of graphene. This difference in thermal conductivity is attributed to the distinct atomic structure and electron configuration of the two materials. The lattice structure of graphene allows for the free flow of phonons, whereas MoS_2_ monolayers have a more complex lattice structure and exhibit phonon scattering [74].

### 2.3. Synthesis Methods of MoS_2_

**Figure 4:**
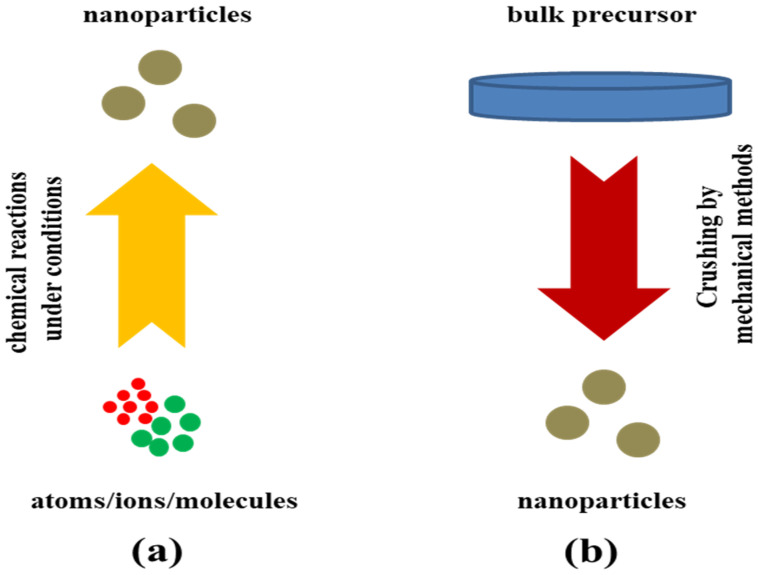
Schematic representation of the (**a**) bottom-up approach and (**b**) top-down approach.

MoS_2_ has emerged as a potential material for advanced portable electronic gadgets like sensors, transistors, and photovoltaic cells in the future. To achieve the desired material features and uses, there are several techniques for synthesizing MoS_2_ and related composites that are more significant. The synthesis of MoS_2_ and its nanocomposites involves top-down and bottom-up methods for fabrication are shown in Figure 4. Chemical vapor deposition (CVD), liquid-chemical exfoliation, hydrothermal synthesis, solvothermal synthesis, and mechanical exfoliation are among the popular methods used for synthesis. The synthesis methodologies for MoS_2_ and its related composites are varied and provide researchers with a range of options for producing high-quality materials for a wide range of applications [75,76,77,78].

#### 2.3.1. Top-Down Approaches

In the top-down method, MoS_2_ bulk crystal is carefully processed to produce MoS_2_ nanomaterials, which are commercially available. This downsizing process involves physical manipulation of the crystal to produce smaller particles with dimensions at the nanometer scale. This method is widely used in nanotechnology and is preferred for its ability to produce uniform and well-defined nanomaterials [79,80,81]. MoS_2_ nanosheets can be readily produced through the exfoliation technique owing to their layered structure and van der Waals forces. There are several exfoliation methods available for synthesizing these nanosheets, such as mechanical, chemical, electrochemical, and liquid-phase exfoliation [82,83,84,85]. Even though the top-down methods are uncomplicated to utilize, they are not successful in producing irregularly shaped and very minute particles. The primary disadvantage of this approach is the challenge of acquiring the appropriate shape and size of particles.

(a) Mechanical Exfoliation

The mechanical exfoliation technique involves the removal of appropriate MoS_2_ flakes from the bulk crystal of MoS_2_ through the use of adhesive tape and then transferring them onto a specific substrate [86,87]. As there is no chemical reaction involved in the mechanical exfoliation of MoS_2_ from its bulk crystal, there will be no alteration in its structure. The exfoliation process yields single- or few-layer MoS_2_ nanosheets with various shapes and sizes after removing the scotch tape, leaving some MoS_2_ residues on the substrate. These nanosheets possess excellent quality, making them ideal for investigating the original material’s properties and device performance. Nevertheless, the yield of production is not high in this process, and it is challenging to regulate the particle size of the resulting MoS_2_ nanosheet [86,88,89]. A study was conducted to investigate the interlayer interactions in heterostructures of transition metal dichalcogenides. The study was successful in creating MoS_2_/WS_2_ and MoSe_2_/WSe_2_ heterostructures using a mechanical transfer technique after exfoliating the relevant crystals [90]. The field of 2D materials has witnessed a major advancement with the creation of an automated approach for exfoliating transition metal dichalcogenides (TMDs) into single-layer or few-layer forms. The technique hinges on deploying controlled normal and shear forces to detach a thin sample from bulk MoS_2_ or MoTe_2_ with the help of a parallel plate rheometer. This detached sample is subsequently affixed to the rheometer’s movable upper fixture through the application of blue dicing tape. The process of creating atomically thin films of specific materials can be initiated through a step-and-repeat exfoliation process by utilizing preprogrammed contact force and liftoff speed. This method results in the consistent formation of such MoS_2_ films [91]. The mechanical exfoliation method is simple, inexpensive, and does not require any special equipment, making it a popular technique for synthesizing high-quality MoS_2_ nanosheets. It can be further optimized by controlling the pressure, speed, and direction of the peeling process to obtain larger and more uniform MoS_2_ nanosheets.

(b) Ball Milling

Ball milling is a mechanical method that involves the use of high-energy ball mills to reduce the size of the precursor materials. The ball milling process has been shown to be a favorable technique for the large-scale production of MoS_2_ nanosheets, as it offers several advantages, such as homogeneity of the final product, low cost, and ease of operation. The procedure can be readily expanded for large-scale manufacturing as well. Additionally, ball milling can be used to produce MoS_2_ nanosheets with varying thicknesses by simply adjusting the milling time and speed [92,93,94]. Cantarella et al. investigated commercial MoS_2_ nanopowders subjected to ball-milling for 20 and 40 h has revealed that the process has significantly enhanced the adsorption properties of the nanopowders. The researchers found that MoS_2_ ball-milled for 40 h showed an exceptional capability to remove methylene blue dye from aqueous solutions. It was able to adsorb all the dye present in just 20 min. This indicates that the milling process led to a considerable increase in the adsorption capacity of the nanopowders [95]. Kumar et al. reported that the ball-milling process was used to treat bulk MoS_2_, resulting in an improvement in the capacitive properties of the material. Specifically, the treated BL-MoS_2_ showed an increase in specific capacitance and energy density, making it highly suitable for use as an active component in supercapacitors [96]. The ultrasonic ball milling technology was utilized for the synthesis of materials like h-BN, graphene, MoS_2_, WS_2_, and BCN. The iron balls used for milling in this study had diameters ranging from 1.0 to 1.5 mm. The outcome achieved was more than 20% in terms of yield. The resulting materials had sizes ranging from 1 to 20 µm and had a thickness of approximately 1–3 nm [97]. Overall, the ball milling process is an effective and efficient method for synthesizing MoS_2_, producing uniform and highly crystalline particles that have potential applications in various fields.

(c) Liquid phase Exfoliation

A popular technique for generating nanosheets and colloidal dispersions involves the use of liquid phase exfoliation (LPE) on layered materials, such as MoS_2_. In this method, the bulk material is dispersed in a solvent to form a suspension and then subjected to high-intensity sonication to break down the layered structure and produce nanosheets. The new approach to producing 2H-MoS_2_ nanosheets, which involves synthesizing two to seven layers, presents several advantages. These advantages include simplicity, effectiveness, environmental friendliness, and absence of contamination. One of the unique aspects of this technique is that no surfactants are required, which can be a common drawback of other methods. Additionally, the use of acetone as a solvent is a notable improvement over NMP, which is commonly used but has low volatility. This technique utilizes solvents that have surface energies that match well with the solid interface, making the synthesis more efficient. By using extended sonication times and low concentrations of bulk powder, the layers are effectively peeled off, and the dispersions remain stable [98,99]. Chlorophyll extracts have been found to be a highly effective exfoliating agent for producing a thin layer of MoS_2_/WS_2_ heterostructure. This novel method of synthesis has opened the door to exciting possibilities in the field of renewable energy. The MoS_2_/WS_2_ heterostructure produced through this method has proven to be an effective catalyst for the production of hydrogen, which is a vital ingredient in a range of clean energy applications [100]. The study examined the electrocatalytic performance of 2H-MoS_2_ nanosheets produced through LPE in N-dimethyl formamide, 1-methyl-2-pyrrolidinone, and formamide by examining their surface and interface functionalization. The findings indicated that MoS_2_ nanosheets produced in formamide were comparatively smaller in size and thickness but showed significantly better electrocatalytic activity when compared to those produced in the other two solvents [101]. One of the biggest challenges in energy research is finding electrode materials for sodium-ion batteries that are inexpensive but can store a large amount of energy. Fortunately, a solution has been found in the form of MoS_2_ nanosheets that are exfoliated in the liquid phase and reinforced with 20 wt% single-wall carbon nanotubes (SWNTs). When shaped into sodium-ion battery electrodes, these materials have impressive gravimetric, volumetric, and areal capacities [102]. The implementation of a partial etching approach has been suggested to be a highly effective method for modifying the thickness and lateral dimensions of MoS_2_ nanosheets, resulting in an increased percentage of edges. This technique has been found to significantly improve the production rate of monolayer MoS_2_ (20%) and WS_2_ (12%) nanosheets through LPE [103].

#### 2.3.2. Bottom-Up Approaches

The bottom-up approach is an effective method for synthesizing MoS_2_ and other 2D materials. The bottom-up approach involves the fabrication of a material from its constituent atoms or molecules. In the case of MoS_2_, it involves the synthesis of Mo and S atoms or molecules, followed by their assembly into the final material. The synthesis process is controlled at the atomic and molecular level, which allows for precise control over the particle size and structure of the resulting MoS_2_ nanocrystals. Bottom-up synthesis methods are highly scalable, making it possible to produce large amounts of MoS_2_ with fewer impurities and defects at a relatively low cost. It requires less energy and fewer processing steps than top-down methods, making them a more energy-efficient and environmentally friendly approach. There are various methods for producing MoS_2_, such as chemical vapor deposition (CVD) and wet chemical synthesis [104].

(a) Chemical Vapor Deposition (CVD)

The preferred method for synthesizing MoS_2_ is chemical vapor deposition (CVD) because it can produce films with large surface areas that have high crystallinity and purity [105]. CVD involves the deposition of MoS_2_ from precursor molecules in the gas phase onto a substrate. It utilizes various methods, including the sulfurization of Mo-based films, thermolysis of S and Mo precursors, and vaporization and decomposition of precursors that contain Mo and S atoms to produce a high-quality film. The thickness of the film can be easily adjusted by varying the duration of the deposition process [106,107,108,109]. The sulfurization of Mo-based films involves the deposition of Mo films followed by the introduction of sulfur gas. The sulfur atoms react with the Mo atoms to form MoS_2._ According to Hong et al., the vapor deposition of sulfurization of MoO_3_ precursor involves three main steps: the adsorption of sulfur on the MoO_3_ surface, the formation of an intermediate sulfur compound, and the final conversion to an MoS_2_ monolayer. The availability of sulfur atoms plays a crucial role in determining the growth rate of the MoS_2_ layer, whereas the quantity of sulfur precursor utilized in the reaction can regulate the thickness of the resulting MoS_2_ layer [110,111]. The oxygen content in the resultant material, specifically MoO_x_S_y_, led to a significant decrease in sheet conductivity. It is important to carefully control the synthesis conditions to minimize the presence of oxygen and other impurities in the material to ensure optimal conductivity and performance [112,113,114].

Rovira et al. reported that the CVD process had successfully synthesized MoS_2_ nanoribbons, where MoO_3_ nanoribbons were used as a template. Two distinct morphologies of MoS_2_ ribbons were obtained by altering the positioning of the crucibles. The first morphology consisted of dense, compact MoS_2_ ribbons, whereas the second morphology comprised ribbons with “thorns” caused by incomplete sulfurization [115]. A new technique called single-step vapor-phase sulfurization has been developed to cultivate uninterrupted films of monolayer MoS_2_. In a study conducted by Chiawchan et al., the process was carried out using MoO_2_ as the source material. The outcome demonstrated a noticeable pattern on the substrate surface, suggesting changes in the concentration-gradient of the Mo-source material throughout the growth phase [116]. The growth of an ordered array of MoS_2_ nanodots with lateral sizes ranging from approximately 100–250 nm has been reported through a thermal chemical vapor deposition process. The process was conducted directly onto SiO_2_ substrates at a relatively low temperature range of 510–560 °C. The results indicate that this CVD method can facilitate the growth of high-quality MoS_2_ nanodot arrays [117]. The integration of the wet synthesis and chemical vapor deposition technique is beneficial for producing MoS_2_ films of superior quality. To create MoS_2_ using the liquid precursor, the process requires transforming a sample coated with a solution of L-cysteine and sodium molybdate while being exposed to an argon gas flow at a temperature of 680 °C. This process results in the direct conversion of the coating into an MoS_2_ film on the substrate [118]. Moreover, the transferring process of prepared films can be quite complicated and may require specialized equipment and expertise. Additionally, achieving uniformity and controllability in the film deposition process can also be a challenge, leading to variability in the quality and properties of the resulting films.

(b) Physical Vapor Deposition

Molecular beam epitaxy (MBE) is a highly advanced technology that has the ability to produce thin films of single-crystal semiconductors. However, its application is limited to the manufacture of two-dimensional (2D) materials [119]. Conversely, physical vapor deposition is not commonly employed for 2D material synthesis. Ti and MoS_2_ materials were employed in the preparation of an MoS_2_/Ti composite using direct current magnetron sputtering. The PVD process is an effective method for growing MoS_2_ at significantly lower temperatures, as low as 350 °C, in this particular study [120]. MoS_2_ can be directly grown on thermally grown silicon oxide and (002) graphite. The technique is based on sputtering and can be easily implemented for growing other TMDs [121]. The electronic and optoelectronic properties of MoS_2(1−x)_Se_2x_ monolayer alloys can be studied through their growth with different edge orientations, specifically the Mo-zigzag and S-Se-zigzag edge orientations. It is possible to obtain these edge orientations by controlling the deposition temperature. Moreover, the remarkably large domain size of Mo_S2(1−x)_Se_2x_ monolayer alloys (x = 0.41–1) can pave the way for the development of high-quality films on a large scale [122]. The study of the early stages of growth of very thin Pd, Au, and Ag films on a single layer of MoS_2_ has uncovered different patterns of growth that were identified via atomic force microscopy. In particular, Pd develops an even layer of contact, whereas Au clusters into small nanostructures, and Ag takes on a scattered formation of islands on the MoS_2_ surface [123].

(c) Wet Chemical Synthesis

Wet chemical synthesis is a commonly employed and economical method for producing few-layered MoS_2_ and related nanocomposites. It involves mixing reagents in a specific order and under certain conditions to control the chemical reactions and the resulting product. The reaction can occur at different temperatures and pressures, and various factors such as pH, concentration, and stirring rate can also affect the final product [124,125]. The hydro/solvothermal method has gained popularity in the hybridization of MoS_2_ with other functional nanomaterials because of its simple procedure, convenient handling, and economical reaction method. Additionally, this method allows for the creation of unique morphologies in metal oxides, hydroxides, and sulfides [126,127]. The ammonium heptamolybdate, MoO_3_, sodium molybdate, etc., are used as precursors for molybdenum and thiourea, sulfur powder, thioacetamide, etc., are used as precursors for sulfur in the preparation of MoS_2_ powder. The hydrothermal/solvothermal methods were widely used for synthesizing a variety of MoS_2_ powder with different structures and properties [128,129,130,131]. The synthesis of MoS_2_ nanosheets and their subsequent aggregation into various morphologies, such as nanotubes, nanoflowers and microspheres etc., holds great potential for applications in diverse fields, including catalysis, energy storage, and bio-imaging [132,133,134].

Luo et al. demonstrated that the preparation conditions of wrapped nano-sheet greatly affect its adsorption capacity. Specifically, a wrapped nano-sheet with a 3:1 ratio of S and Mo, hydrothermally treated at 240 °C for 37 h, exhibited the highest adsorption capacity. The gradual evolution of morphology from coral-like aggregates to flower-like spheres to wrapped nano-sheet structures is significant as it enables the production of three distinct morphologies that can cater to varying demands in various fields [135]. The production of MoS_2_ nanosheets has been accomplished effectively through an uncomplicated and environmentally friendly hydrothermal technique that utilizes l-cysteine and ammonium molybdenum hydrate. During the synthesis process, l-cysteine can serve a dual purpose as a sulfur source and as a capping agent [136]. MoS_2_ nanosheets with different layer thicknesses (bilayer, trilayer, quadrilayer, and more) and a combination of MoS_2_ quantum dot-nanosheets were produced using a hydrothermal process by controlling the amount of MoS_2_ and NaOH. NaOH serves as an agent for intercalating, and the fragmentation of nanosheets results in the production of quantum dots [137]. The study conducted by Cordeiro et al. highlights the possibility of utilizing microwave-assisted hydrothermal synthesis to cultivate MoS_2_ nanostructures on cellulose paper substrates. This development presents potential applications in the fields of electronics and sensors. The researchers investigated the influence of synthesis parameters like temperature and time on the quantity of MoS_2_ grown on cellulose fibers. The evaluation identified the existence of both metallic and semiconductor phases of the nanostructures [138]. Xuan et al. investigated the effect of reaction temperature (160–220 °C) and reaction time (6–14 h) on the microstructure and morphology of MoS_2_. The outcomes showed that as the reaction heat increased from 160 to 220 °C, the morphology of MoS_2_ transformed gradually from aggregated particles to flower-like spheres to a nanosheet structure. In contrast, the reaction time affected the restacking and refinement of the MoS_2_ crystal structure but did not have a significant impact on the morphology [139]. Duraisamy et al. described a straight forward hydrothermal method for creating a 3D network of metallic MoS_2_/MoO_3_ nanosheets. The study found that the resulting nanosheets exhibit not only the desired metallic MoS_2_ phase but also a significant density of active sites and a tuned electrical conductivity that contribute strongly to their catalytic activity and hydrogen evolution reaction [140]. The comparison of advantages and disadvantages of various synthesis methods are listed in Table 1.

## 3. Energy Applications

**Figure 5:**
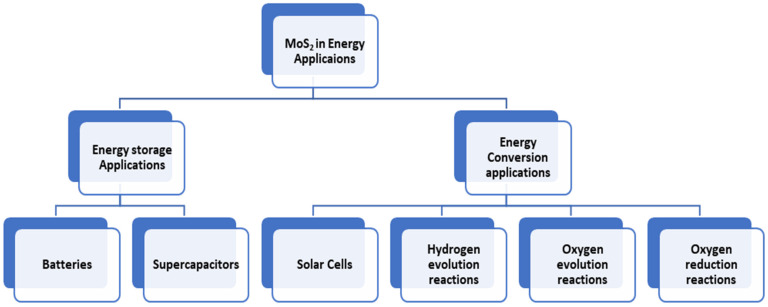
Various applications of MoS_2_ in energy storage and energy conversion fields.

MoS_2_ shows great potential for use in energy-related applications. However, further research and developments are necessary to address the scalability, stability, and cost-effectiveness challenges associated with its integration into the energy industry. We will categorize our conversation about energy applications into two primary domains, namely, the storage and conversion of energy. Ultimately, we will examine the challenges that MoS_2_ might encounter during its integration into the energy sector [141,142]. Various applications of MoS_2_ in energy storage and conversion fields are shown in Figure 5.

### 3.1. MoS_2_-Based Nanocomposites in Energy Applications

MoS_2_ composites have gained significant attention in recent years due to their unique properties that make them useful in energy applications. Here are some reasons why MoS_2_ composites are important in these applications:

1. Improved conductivity: MoS_2_ composites offer improved electrical conductivity compared to pure MoS_2_, making them ideal for use in batteries, fuel cells, and other energy storage and conversion devices;
2. Enhanced catalytic activity: MoS_2_ composites exhibit enhanced catalytic activity, which is useful in energy conversion processes like hydrogen evolution reaction (HER), oxygen reduction reaction (ORR), and carbon dioxide reduction reaction (CO_2_RR);
3. Increased stability: MoS_2_ composites are more stable than pure MoS_2_ and other transition metal dichalcogenides (TMDs) under extreme conditions, such as high temperatures and corrosive environments. This makes them suitable for use in harsh industrial and energy-related applications;
4. Reduced cost: MoS_2_ is a low-cost material, and its composites can be produced by simple, scalable processes, making them an economical option for energy applications.

In conclusion, MoS_2_ composites play a crucial role in energy production, storage, and conversion, making them highly useful in energy applications. With their exceptional properties, they have opened up new avenues for the development of highly efficient and sustainable energy technologies.

### 3.2. Energy Storage Applications

MoS_2_ is a potential material for energy storage applications due to its remarkable ability to store high amounts of energy and its extended lifespan. Its potential has been explored for its utilization in energy storage applications, specifically supercapacitors, sodium-ion batteries (SIBs) and lithium-ion batteries (LIBs). MoS_2_ nanosheets can also improve the overall performance of LIBs by improving their specific capacity and cycle life. MoS_2_ has displayed positive outcomes as a cathode material in SIBs concerning long cycle life and high energy density. Similarly, it has been observed that MoS_2_ carries a high surface area, excellent electrical conductivity, and exceptional electrochemical stability, making it an ideal electrode material for supercapacitors. Studies have revealed that MoS_2_-based supercapacitors exhibit better energy storage capacity than others, high power density, and long cycle life [143,144,145,146,147,148].

#### 3.2.1. Li-Ion Batteries

**Figure 6:**
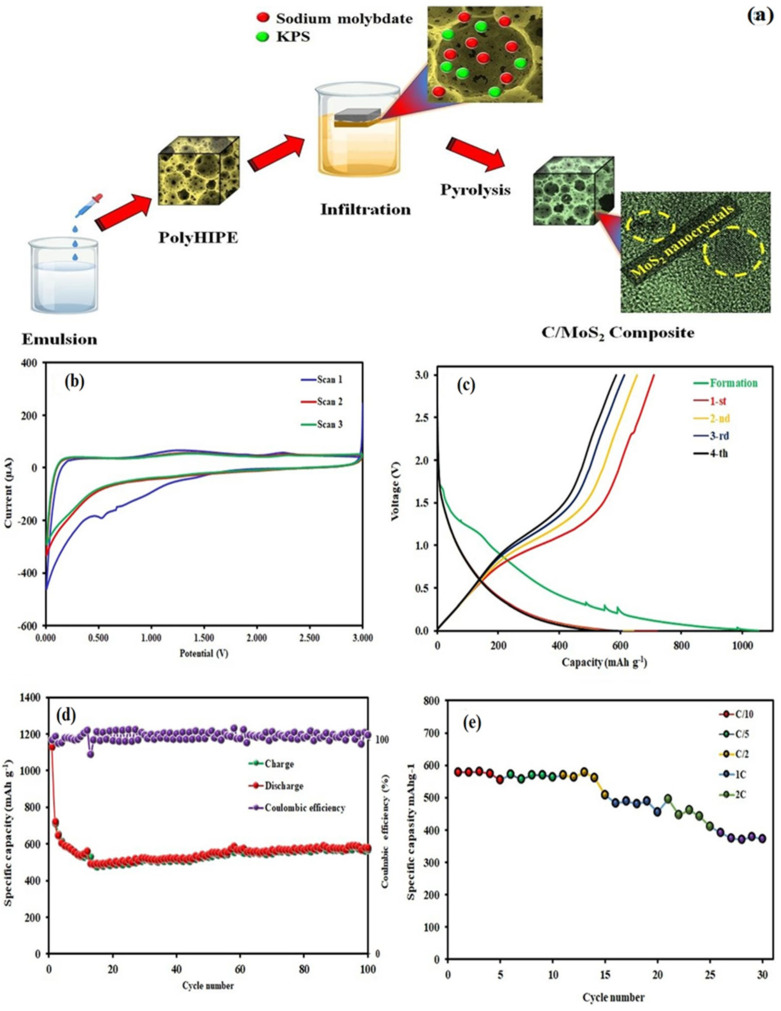
(**a**) Schematic representation of the synthesis of C/MoS_2_ hybrid, (**b**) CV curves of C/MoS_2_ electrode at a scan rate of 1 mV s^−1^ within a potential window of 0.01–3.0 V, (**c**) GCD curves within a potential window of 0.01–3.0 V (vs. Li/Li+), (**d**) Cyclic performance of C/MoS_2_ electrode, and (**e**) Rate capability of C/MoS_2_ electrode. Adopted from [151]. Copyright 2023 Scientific Reports.

MoS_2_ has become a popular material for lithium-ion batteries (LIBs) in recent times. This is because it possesses distinctive characteristics that can enhance the performance and durability of the batteries. MoS_2_ acts as an anode or cathode in Li-ion batteries. It has a high inherent capacity for storing lithium ions, the ability to prevent lithium-ion insertion/extraction during charge cycles, and stability over long-time cycling. It has been shown that by incorporating MoS_2_ as an electrode into Li-ion batteries, the battery life can be increased due to the superior charge/discharge properties and stability of the MoS_2_ material [149]. Liu et al. introduced a new MoS_2_/C hybrid electrode for use as a Li-ion battery anode. This electrode exhibits impressive low-temperature performance, maintaining a stable discharge capacity of up to 854.3 mAh g^−1^ at −20 °C, which is equivalent to 72.8% of its room-temperature performance. Despite operating at a current density of 3 A g^−1^, this electrode continues to exhibit a capacity of 140.9 mAh g^−1^. The increased spacing between MoS_2_ layers promotes rapid Li+ diffusion, ultimately enhancing the kinetics of lithiation/delithiation and improving the performance rate at low temperatures [150]. Baheri et al. developed an innovative method to produce 1T/2H-MoS_2_ nanocrystals within N-doped nanoporous carbon shown in Figure 6. This process involves using a pyrolysis method of sodium molybdate and potassium persulfate within an acrylonitrile-based polymer base. As a result, the C-MoS_2_ composite showed exceptional battery performance, with a specific capacitance of 556 mAh g^−1^ at a current density of 0.2 A g^−1^ and maintained excellent stability even after undergoing 100 cycles [151]. A recent study has shown that a 3D bulky MoS_2_@C/RGO composite may offer significant benefits for the cycling performance and rate capability of Li-ion batteries. The synthesized nanocomposite demonstrated a comparable cycling performance of 1189 mAh g^−1^ after undergoing 100 charge/discharge cycles at 200 mA g^−1^, which was better than the control samples and earlier reports [152]. The composite also showcased a good rate capability, with 1211 mAh g^−1^ at a current density of 50 mA g^−1^ and even 726 mAh g^−1^ at a current density of 2000 mA g^−1^. Moreover, when tested in an MoS_2_-PDA-GO30//LiCoO_2_ full-cell configuration, this composite material exhibited a superior cycling capability of 890 mAh g^−1^, which is equivalent to 1750 Wh kg^−1^. Despite the challenges, MoS_2_ remains a promising electrode material for LIBs, and research efforts are ongoing to address its cycle stability issues [153]. The widespread use of MoS_2_ electrodes in LIBs could potentially lead to the development of more efficient and longer-lasting batteries, with significant implications for various industries and applications. Bozheyev et al. investigated the use of MoS_2_ nanopowder as an anode material in LIBs. The nanopowder was produced through a solid-state synthesis method (SHS). The electrochemical performance of this nanopowder was evaluated through a coin-type CR2032 cell. According to the findings, MoS_2_-NP displayed an initial charging ability of 567 mAh g^−1^ when subjected to a current density of 50 mAh g^−1^. This indicates that MoS_2_-NP could be a promising contender for a proficient anode substance in LIBs [154]. The study on MoS_2_ phase transformation and the incorporation of CNT additives in batteries have showcased promising results in the application of metal sulfide electrodes in high-energy-density batteries [155].

The MoS_2_ composite electrodes were prepared by a method of uniaxial cold-pressing, which involved the pressing of the materials under a high pressure of 3 tons. The composite electrodes contained 60 wt% MoS_2_, 10 wt% carbon black, and 30 wt% β-Li_3_PS_4_ solid electrolyte. The solid electrolyte used in these electrodes was β-Li_3_PS_4_, which has high ionic conductivity and stability in air. Solid-state lithium-ion batteries were utilized to assess the efficiency of these electrodes, with a Li-In alloy serving as the counter electrode. During the discharge process at a rate of 67 mA g^−1^ (C-10) within the voltage range of 0.01–3.0 V, the electrodes displayed an initial specific capacity of approximately 439 mAh g^−1^. The specific capacity retention was also outstanding, with 312 mAh g^−1^ obtained after undergoing 500 cycles [156]. MoS_2_ and its composites have garnered increasing attention as potential electrode materials in the field of battery technology. One key advantage is their larger interlayer spacing, which enables fast metallic ion diffusion compared to other materials. This is particularly useful for ions like Na^+^, Zn^2+^, and Mg^2+^, which are commonly used beyond LIBs. Furthermore, the usual abundance of molybdenum and sulfur means that MoS_2_-based electrodes can be produced at a relatively low cost compared to other materials [157]. Cha et al. demonstrated that the incorporation of MoS_2_ in Li metal batteries can effectively mitigate the issues of dendrite formation and electrolyte decomposition, which are notorious for causing safety hazards and performance degradation. MoS_2_ can be used as a protective coating for Li metal anodes to improve their cyclic stability and avoid the formation of lithium dendrites. The use of MoS_2_ as a separator modification can also improve the battery’s performance by enhancing the wettability of the electrolyte and decreasing the impedance. Moreover, the solid electrolyte interphase formed between MoS_2_ and Li metal can serve as a stable interfacial layer, reducing the risk of side reactions between the electrolyte and Li metal [158].

#### 3.2.2. Sodium-Ion Batteries

Molybdenum-based materials have shown remarkable electrochemical properties and stability, making them a favorable choice for anode materials in sodium-ion batteries (SIBs). These materials have been researched in several different forms, like nanoparticles, flakes, and membranes. They have demonstrated encouraging outcomes regarding their ability to maintain capacity and stability during cycles. One of the key advantages of molybdenum-based materials is their ability to interact with sodium ions through various mechanisms, including alloying, intercalation, and conversion reactions. The unique Na-insertion properties of molybdenum-based materials can be attributed to the open framework of their crystal structure, which can accommodate large sodium ions and allow for reversible and sustainable storage [159]. A combination of two distinct 1T and 2H phases has been utilized to produce a dual-phase MoS_2_ (DP-MoS_2_). This electrode has displayed exceptional cycling stability, with a reversible capacity of 300 mAh g^−1^ after undergoing 200 cycles, as well as excellent rate capability with a capacity of ~220 mAh g^−1^ at 2 A g^−1^. These findings indicate that DP-MoS_2_ has the capability to be utilized in commercial devices, offering a fresh strategy to create metal chalcogenides for applications in storing electrochemical energy [160]. A thorough analysis of MoS_2_ in comparison with MoO_2_ indicates that MoS_2_ has the ability to store sodium through the insertion reaction due to its larger interlayer distance compared to MoO_2_. Furthermore, the weaker bonds of MoS_2_, as compared to MoO_2_, make the conversion reaction of sodium with MoS_2_ more favorable [161]. The MoS_2_/grapheme/chitosan electrode has demonstrated superior performance as an anode material for SIBs. After undergoing 200 charge/discharge cycles at a current density of 100 mA g^−1^, the electrode exhibited a high capacity retention of 527.3 mAh g^−1^. It also displayed excellent rate capability and maintained a high capacity of 439.1 mAh g^−1^ at 1 A g^−1^. Importantly, the MoS_2_/G/C electrode exhibited exceptional stability over a long cycle life span, making it a favorable material for high-performance SIBs [162]. MoS_2_@CNTs composite material has been found to exhibit remarkable performance as an anode material for SIBs. This composite material demonstrates a high rate of performance and can deliver 508, 418, 359, 305, 258, and 183 mAh g^−1^ at current densities of 0.1, 0.2, 0.5, 1, 2, and 5 A g^−1^, respectively. The remarkable cycling stability of this composite material allows it to retain 360 mAh g^−1^ after undergoing 400 charge/discharge cycles at 0.5 A g^−1^. The outstanding ability of MoS_2_@CNTs is attributed to the strong combination of the MoS_2_ and CNT components, which makes it highly robust [163].

**Figure 7:**
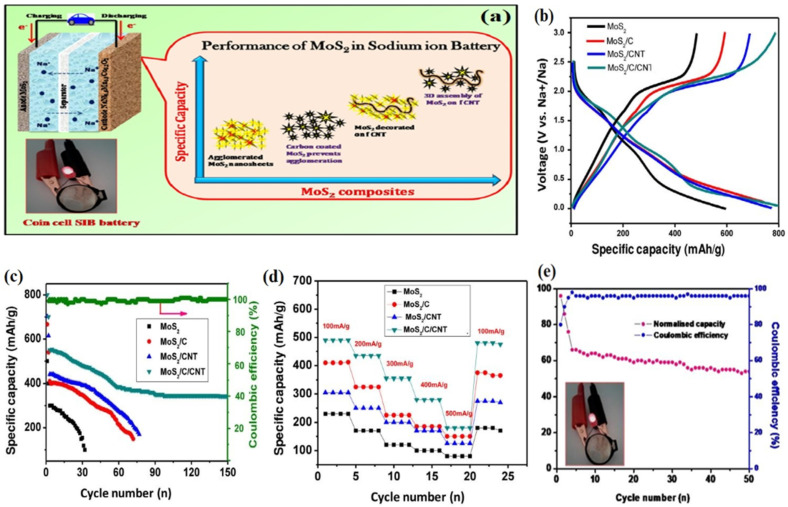
Electrochemical performance of MoS_2_, MoS_2_/C, MoS_2_/CNT, and MoS_2_/C/CNT electrodes in sodium-ion batteries: (**a**) schematic representation of the performance of MoS_2_ and its composites in sodium-ion batteries, (**b**) GCD characteristics of MoS_2_ and its composites at 50 mA g^−1^, (**c**) cyclic test of MoS_2_ and its composites at 50 mA g^−1^ (the green arrow indicates that MoS_2_/C, MoS_2_/CNT composite electrode have high cyclic stability), (**d**) cyclic rate of MoS_2_ and its composites at various current densities, and (**e**) cycling performance MoS_2_/C/CNT//Na(Ni_0.5_Mn_0.3_Co_0.2_)O_2_ full-cell at 50 mA g^−1^. Adopted from [167]. Copyright 2021 Carbon Trends.

Ye et al. offer a different approach to enhancing the cycling performance of SIBs with respect to their reversible capacity. The remarkable performance of the Metal-semiconductor phase twinned hierarchical (MPTH) MoS_2_ electrode is ensured by expanding interlayers and preserving structural stability. It has been proven to deliver high reversible capacities of up to 200 mAh g^−1^ at a current density of 0.1 A g^−1^ for undergoing 200 cycles and an impressive 154 mAh g^−1^ at a current density of 1 A g^−1^ for undergoing 2450 cycles in the voltage range of 0.4–3.0 V. The electrode is capable of maintaining 6500 cycles when exposed to a current density of 2 A g^−1^, which results in 82.8% capacity retention from the second cycle. These findings exceed all previously recorded cycling performances of MoS_2_ by a significant margin [164]. A novel method for producing molecule-intercalated MoS_2_ with accurate interlayer spacing ranging from 0.62 to 1.24 nm has been suggested in a recent research study. This innovative technique allows for the fine-tuning of electrical conductivity, which ranges from 1.3 × 10^−4^ to 3.5 × 10^−2^ S cm^−1^, through incorporating guest molecules into MoS_2_. The synthesized MoS_2_ exhibits excellent sodium ion storage capacity, with the highest initial capacity of 465 mAh g^−1^ and retaining a capacity of 420 mAh g^−1^ after undergoing 600 charge/discharge cycles at a rate of 100 mA g^−1^. The better electronic conductivity and increased interlayer spacing collaborate to produce a synergistic impact that promotes the rapid movement of sodium ions and electrons. As a result, material usage is more efficient, and the outcomes are impressive [165]. Chothe et al. proposed a technique based on the solid-state method to generate ultrathin nanosheets of MoS_2_-graphene, which can be utilized in Na-ion batteries. During the galvanostatic study, the assembled Na-ion cell demonstrated a high specific capacity of 420 mAh g^−1^ at 50 mA g^−1^ and a capacity retention of 172 mAh g^−1^ at 200 mA g^−1^ after undergoing 200 cycles. This study highlights the potential of using hybrid materials in battery applications and demonstrates the importance of optimizing the electrode architecture for improved battery performance [166]. Halankar et al. developed an MoS_2_/C/CNT nanocomposite using a simple hydrothermal method shown in Figure 7. This composite material served as an electrode in SIB applications. The composite possesses excellent electronic conductivity and mechanical strength due to the presence of CNT, and at the same time, it effectively captures the polysulfide forms that occur during cycling. The full-cell SIB was developed using Na(Ni_0.5_Mn_0.3_Co_0.2_)O_2_ as a positive electrode and MoS_2_/C/CNT as a negative electrode. This battery demonstrated a specific capacitance of 96 mAh g^−1^ at a current density of 50 mA g^−1^ [167]. Researches on MoS_2_ in Na-ion batteries are still in its early stages, but there is potential for this material to show a significant role in the improvement of next-generation energy storage devices. Further investigation and optimization of the material’s properties and performance will be crucial for its successful integration into commercial Na-ion batteries.

#### 3.2.3. Supercapacitors

MoS_2_ has a two-dimensional layered structure with remarkable electrochemical activity. This characteristic makes it an excellent option to serve as an electrode substance in supercapacitors, which require materials with high surface area and low resistance to store and release electrical energy quickly. Additionally, MoS_2_ has a high capacitance, which makes it an attractive option for supercapacitors, as increased capacitance typically leads to higher energy storage capacity.

(a) 1T and 2H MoS_2_ Electrodes for Supercapacitors

The surface functionalization of 1T MoS_2_ nanosheets with NH_2_Ph ring, through the chemical reaction with iodobenzene, leads to the formation of Fc-1T MoS_2_, which exhibits improved electrocatalytic performance as compared to 1TMoS_2_. By adding NH_2_Ph ring functionality and 1T MoS_2_ phase, the charge density and electronic properties are improved, resulting in faster charge transport and greater charge storage capacity. Fc-1T MoS_2_ displays a significant specific capacitance of 501 F g^−1^ at 1 A g^−1^ while maintaining 76.32% capacitance retention even after 2000 cycles. This is 2.6 times greater than the bare 1T MoS_2_ nanosheets [168]. The latest combination of ammonium ion intercalation molybdenum disulfide (A-MoS2) with a considerable 1T proportion exhibits great potential in augmenting the functioning of energy storage devices, such as supercapacitors and batteries. Its substantial specific capacitance of 228 F g^−1^ at 5 mV s^−1^ indicates that A-MoS_2_ possesses a remarkable charge-storing capacity. Additionally, A-MoS_2_’s retention capability is also noteworthy, retaining 127 F g^−1^ at 80 mV s^−1^, which is vital for real-world usage [169]. Zarach et al. demonstrate the potential of Pt@1T/2H-MoS_2_ electrode material for energy storage applications, as it shows significantly improved specific capacitance and consistent coulombic efficiency during multiple charging and discharging cycles. The surface modification with Pt shows a crucial role in enhancing the material’s electrochemical properties by promoting the exfoliation process [170]. Shrivastav et al. synthesized 2H-MoS_2_ using a hydrothermal method [171]. At a scanning rate of 10 mv s^−1^, 2H MoS_2_ demonstrated a specific capacitance of 115.42 F g^−1^, and at a current density of 0.5 A g^−1^, it showed a specific capacitance of 57.69 F g^−1^. The 2H MoS_2_ also exhibited an electrochemical double-layer capacitive behavior with partial pseudo-capacitance. The retention of 71.5% of specific capacitance after 5000 CV cycles indicates good cyclic stability of the samples.

(b) MoS_2_ with Graphene and Carbon-Based Nanocomposites

MoS_2_ with graphene-based nanocomposites have been researched as electrode materials for supercapacitors owing to their exceptional electrical conductivity and high surface area [172]. Moreover, the addition of carbon-based materials like carbon nanotubes or carbon black can enhance the electrochemical activities of the composite, improving the overall performance of the supercapacitor [173,174]. Ramakrishnan et al. developed an MoS_2_@rGO nanocomposite through a hydrothermal synthesis followed by an ultra-sonication method. This composite has been shown to be a profound candidate for sodium ion symmetric hybrid SCs. The electrode material was examined in a half-cell formation using metallic sodium and exhibited a high specific capacity of 226 F g^−1^ at a current density of 0.03 A g^−1^. In a full-cell configuration, the electrode exhibited a high capacitance of 55 F g^−1^ at a current density of 0.03 A g^−1^ [175]. Zhan et al. developed high-performance hybrid supercapacitors (HSCs) based on an interlayer-expanded MoS_2_/rGO nanocomposite. The sodium hybrid supercapacitor (Na-HSC) outperforms the lithium hybrid supercapacitor (Li-HSC) when combined with N-doped hierarchically porous 3D graphene (N-3DG). It displays remarkable performance, achieving an energy density of 140 Wh kg^−1^ at a power density of 630 W kg^−1^ and an energy density of 43 Wh kg^−1^ at an extremely high power density of 103 kW kg^−1^ (with a charge completed within 1.5 s). Furthermore, even after over 10,000 cycles, there is no apparent reduction in capacity [176]. Ji et al. synthesized an MoS_2_-reduced graphene oxide (rGO) in a sandwich structure through a solvent-free and one-step ball-milling process. The resulting MoS_2_-rGO nanocomposite sample exhibited exceptional electrochemical performance when used as an electrode material for supercapacitors. The composite demonstrated a high specific capacitance of 306 F g^−1^ at a current density of 0.5 A g^−1^, which was higher than that of pure MoS_2_. Additionally, the composite exhibited minimal discharge capacity decay after 100 cycles in li-ion batteries (0.2 A g^−1^) and 5000 cycles in supercapacitors (4 A g^−1^), demonstrating its excellent potential for long-term energy storage devices [177]. Quan et al. discovered a novel nanostructure composed of hollow carbon–MoS_2_–carbon, which has been synthesized using an l-cysteine-assisted hydrothermal method. The hollow nanoplate morphology of the material provided a high specific surface area of 543 m^2^ g^−1^ and a total pore volume of 0.677 cm^3^ g^−1^, with fairly small mesopores at approximately 5.3 nm. These properties make the hollow carbon–MoS_2_–carbon nanoplates ideal for use in supercapacitors. When tested in a symmetric supercapacitor, the material exhibited a specific capacitance of 248 F g^−1^ (0.12 F cm^−2^) at a constant current density of 0.1 A g^−1^ [178]. The MoS_2_/CNT composite is a highly effective material for producing high-performance supercapacitors. At a current density of 1 A g^−1^, the MoS_2_/CNT composite has a specific capacitance of 402 F g^−1^. This is an impressive result that indicates high energy storage capacity. Moreover, the composite maintains 81.9% capacitance retention after 10,000 continuous charge/discharge cycles at the same high current density, indicating excellent cycling stability [179].

Mahajan et al. successfully developed a high-performance supercapacitor using biocarbon-based MoS_2_ nanoparticles synthesized from date fruits. By converting agricultural biowaste into an easily affordable energy resource via the pyrolysis technique, the team has developed a carbon-based nanocomposite that exhibits a high specific capacitance of 945 F g^−1^ at a current density of 0.5 A g^−1^. This biocompatible Bio-C/MoS_2_ nanocomposite displayed an impressive reproducing stability of 92% even after 10,000 charge/discharge cycles. Furthermore, the Bio-C/MoS_2_ nanosphere demonstrated an exceptionally high power density of 3800–8000 W kg^−1^ and an energy density of 74.9–157 Wh kg^−1^, making it a promising material for high-performance energy devices [180]. Zhang et al. reported the successful development of a new hierarchically porous MoS_2_/C composite aerogel using a simple one-pot mass preparation method. The composite aerogel displays a high specific capacitance of 712.6 F g^−1^ at 1 A g^−1^ and a capacity retention rate of 97.3% after 13,000 cycles at 6 A g^−1^ when tested as a supercapacitor electrode [181].

(c) MoS_2_ with Metal Oxide/Sulfide-Based Nanocomposites

The electrochemical performance of MoS_2_ is restricted by its low surface area and porosity. To enhance its performance, nanocomposites can be produced using metal oxides or sulfides, which have high surface areas and better ionic conductivity. The use of nanocomposites combining MoS_2_ with metal oxides or sulfides shows significant promise for improving the performance of supercapacitors. The study conducted on CoNi_2_S_4_ and an MoS_2_ nanocomposite fixed on porous graphene oxide yielded promising results in their potential as a high-performance substrate. The spongy nanocomposite showed a remarkable specific capacitance of 3268 F g^−1^ in a 3 M KOH solution at 1 A g^−1^. Furthermore, the CoNi_2_S_4_@MoS_2_@rGO electrode displayed an impressive 93.6% stability of its initial capacity after 3000 consecutive charge/discharge cycles at a current density of 10 A g^−1^. Moreover, the energy density of 41 Wh kg^−1^ and power density of 700 W kg^−1^ demonstrate the potential for this electrode to operate effectively under high power needs, indicating its wide-ranging applicability in supercapacitor applications [182]. The Bi_2_S_3_/MoS_2_ nanohybrid, which has a flower-like structure and contains high-quality heterostructures and 3D aisles, boasts exceptional electrochemical capabilities. This includes a high specific capacitance of 1258 F g^−1^ at 30 A g^−1^ and outstanding cycle stability of 92.65% after 5000 cycles at 10 A g^−1^ [183]. The prepared SnS_2_/MoS_2_ layered heterojunction has shown remarkable promise in terms of supercapacitor properties when compared with the sole SnS_2_. The specific capacitance achieved was a noteworthy 466.6 F g^−1^ at a current density of 1 A g^−1^, indicating a significant improvement. Furthermore, the experiment demonstrated impressive cycling stability, with 88.2% capacitance retention at a current density of 4 A g^−1^. The as-fabricated symmetric supercapacitor displayed an energy density of 115 Wh kg^−1^ at a power density of 2230 Wh kg^−1^, which demonstrates its high energy storage capacity in high power demand situations [184]. Govindan et al. highlighted the exceptional properties of CeO_2_/C/MoS_2_ hybrid for charge storage applications. The hybrid exhibited an outstanding specific capacitance of 1325.67 F g^−1^. This value is significantly higher than that of CeO_2_/C (727.49 F g^−1^) or MoS_2_ (300.33 F g^−1^) alone. Furthermore, the cyclic stability of the hybrid was excellent, as it showed a capacitance retention of 92.8% after 1000 charging/discharging cycles. The asymmetric supercapacitor (ASC) showed a remarkable electrochemical performance with a high specific capacitance of 110.55 F g^−1^, excellent cyclic stability (even after 1000 cycles) and a high energy density of 34.55 Wh kg^−1^ at a power density of 666.7 W kg^−1^ [185].

**Figure 8:**
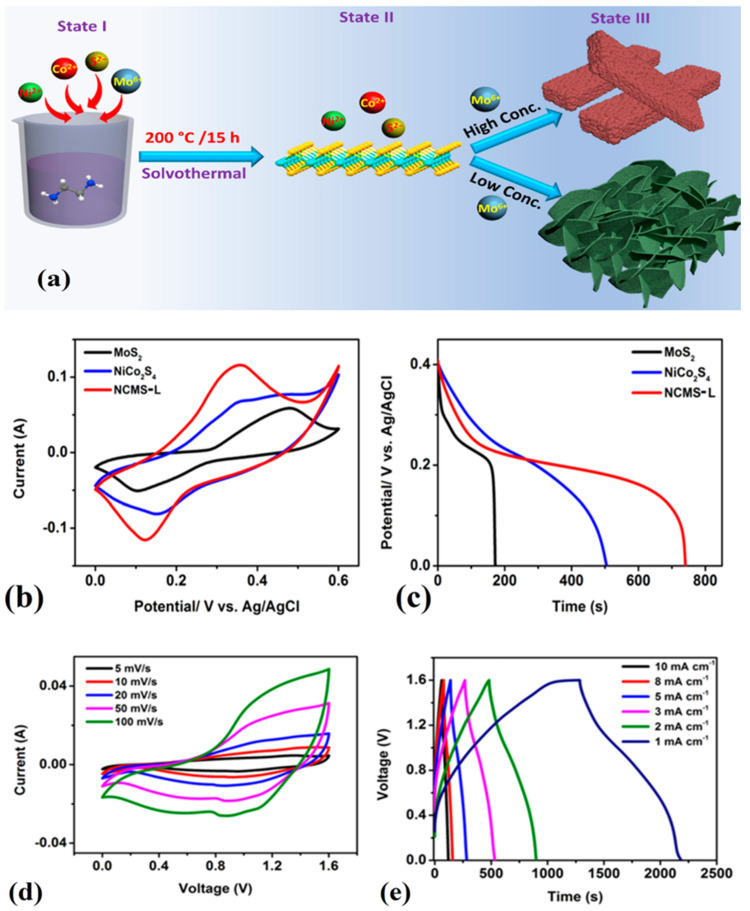
(**a**) Schematic representation of the synthesis process of NiCo_2_S_4_/MoS_2_ composite (State I: shows the mixing of all precursors, State II: illustrates the formation of MoS_2_, State III: demonstrates the formation of NiCo_2_S_4_ over MoS_2_ with sheet- and rod-like morphologies), (**b**) Comparative CV curves of MoS_2_, NiCo_2_S_4_, and NCMS-L (NiCo_2_S_4_/MoS_2_ composite with sheet-like morphology) at a scan rate of 100 mV s^−1^, (**c**) Comparative GCD curves of MoS_2_, NiCo_2_S_4_, and NCMS-L at a current density of 1 mA g^−1^, (**d**) CV curves of the hybrid supercapacitor device of NCMS-L and activated carbon at various scan rates, (**e**) GCD curves of the hybrid supercapacitor device of NCMS-L and activated carbon at various current densities. Adopted from [187]. Copyright 2023 Nanomaterials.

The preparation of an MoS_2_/Fe_2_O_3_/G composite heterostructure material through the hydrothermal method has enabled the development of a highly efficient electrode material for supercapacitor devices. The unique combination of MoS_2_, Fe_2_O_3_, and graphene has resulted in a significant increase in capacitance, with a value of 98.2 mAh g^−1^ at 1 A g^−1^. Importantly, the ASC device incorporating the MoS_2_/Fe_2_O_3_/G composite electrode has shown remarkable energy and power density of 46.8 Wh kg^−1^ and 750 W kg^−1^, respectively. Moreover, the impressive performance of the device is retained over a prolonged duration of usage with excellent capacity retention of up to 77% even after 10,000 cycles [77]. MnS/MoS_2_/Ni-Inverse opal structure nanocomposites were synthesized using the electrochemical deposition (ECD) technique. These specimens demonstrated exceptional specific capacitance values of 1880 F g^−1^ at a charge current density of 5 A g^−1^. Furthermore, the samples were subjected to 2000 cycles of cycle life testing, and even after this rigorous testing, they retained a respectable capacitance value of 76.6% of their initial value [186]. Tue et al. proposed a novel method to produce NiCo_2_S_4_/MoS_2_ composite with different morphologies (like sheet and rod shape) using a simple solvothermal method shown in Figure 8. The composite electrode in sheet-like morphology (NCMS-L) demonstrated remarkable performance, including outstanding coulombic efficiency, elevated specific capacitance, exceptional energy density, and good cyclic stability. Specifically, the electrode presented a capacity of 2594 F g^−1^ at a current density of 0.8 A g^−1^ after undergoing 45,000 charge and discharge cycles. Additionally, the hybrid supercapacitor device comprising NCMS-L and activated carbon achieved an energy density of 31.9 Wh kg^−1^ [187].

(d) MoS_2_ with Conducting polymer-based nanocomposites

In recent years, conducting polymers have gained increasing attention as electrode materials because of their numerous benefits. One of the most significant benefits of using conducting polymers is their low cost compared to traditional electrode materials such as metals. Additionally, conducting polymers possess high electrical conductivity, providing efficient transport of charge between the electrode/electrolyte interfaces. Chen et al. synthesized a hybrid electrode material, MoS_2_@PANI, by using a one-pot hydrothermal synthesis method. The resultant material had 25 wt% MoS_2_ and exhibited a high capacitance of 645 F g^−1^ at a current rate of 0.5 A g^−1^, which indicates maximum energy storage capacity. Additionally, this hybrid electrode material showed good cycling stability with a retention rate of 89% of its capacitance value after 2000 cycles at a current density of 10 A g^−1^ [188]. The CeO_2_/MoS_2_/PANI nanocomposite has demonstrated impressive electrochemical functionality, as evidenced by its specific capacitance values of 177 F g^−1^ at a scan rate of 5 mV s^−1^ and 62 F g^−1^ at a current density of 0.5 A g^−1^. This indicates its ability to store electrical charge efficiently. Additionally, the composite has exhibited excellent cyclic stability, with a capacitance retention rate of 98% after 2000 cycles at a current density of 5 A g^−1^, indicating its durability and long lifespan [189]. Ren et al. successfully developed MoS_2_/PANI nanocomposites with varying PANI loadings, using MoS_2_ nanosheets as the substrate. The composites with a 53 wt% PANI loading displayed the highest electro-capacitive property, resulting in exceptional electrochemical performance with a symmetric supercapacitor having an energy density of 35 Wh kg^−1^ and a power density of 335 W kg^−1^. Furthermore, the material demonstrated excellent cycling stability, showing 81% retention after 8000 cycles [190]. The preparation of a highly efficient MoS_2_/PANI/functionalized carbon cloth (MoS_2_/PANI/FCC) has been accomplished through a drop-casting method. This method allows for facile construction of the MoS_2_/PANI/FCC-based symmetric supercapacitor, which demonstrates a specific capacity of 72.8 F g^−1^ at 0.2 A g^−1^, based on the total mass of the two electrodes with a retention rate of 85% after 1000 cycles [191].

(e) MoS_2_ based Flexible and wearable Supercapacitors

The emergence of wearable and flexible energy storage systems has revolutionized the field of wearable technology. These energy storage systems are designed to provide a reliable and sustainable source of power to wearable devices, including medical monitors, fitness trackers, and smartwatches. They are ultra-thin and flexible, which allows them to be integrated into the fabric of clothing or the band of a watch, making them easy to wear and practically invisible. The capability to deliver consistent and dependable power is a crucial factor for any wearable energy storage gadget. These devices must be able to store enough energy to last for an extended period, and they must also be capable of delivering a high current rate to meet the demands of different wearable devices. To achieve this level of performance, a variety of energy storage technologies are being researched, including batteries, supercapacitors, and hybrid systems that combine the best of both worlds [192,193,194]. Over the past few years, the wearable electronics industry has shown a remarkable interest in two-dimensional nanomaterials such as graphene, transition metal dichalcogenides (TMDs), and black phosphorus. This is because they possess distinctive features and benefits when compared to conventional materials [195,196,197,198,199,200,201]. One of the main benefits of these materials is their layer thickness, which is typically only a few atoms thick, making them ultra-thin and lightweight. In addition, the weak van der Waals force between layers enables simple exfoliation of these materials into thin layers, which enables the development of thin layers exhibiting distinct properties [202]. Due to its remarkable electrical conductivity, high surface area, and chemical stability, MoS_2_ has become a prominent contender as a material for flexible supercapacitor electrodes. When employed as an electrode material in flexible supercapacitors, MoS_2_ can improve their energy storage capacity, as well as their overall stability. The utilization of MoS_2_ in these capacitors shows great potential for next-generation energy storage devices that are lightweight, adaptable, and highly effective. Continued research into the fabrication and optimization of MoS_2_-based supercapacitors will be critical for realizing these capabilities and advancing the field of energy storage [203].

**Figure 9:**
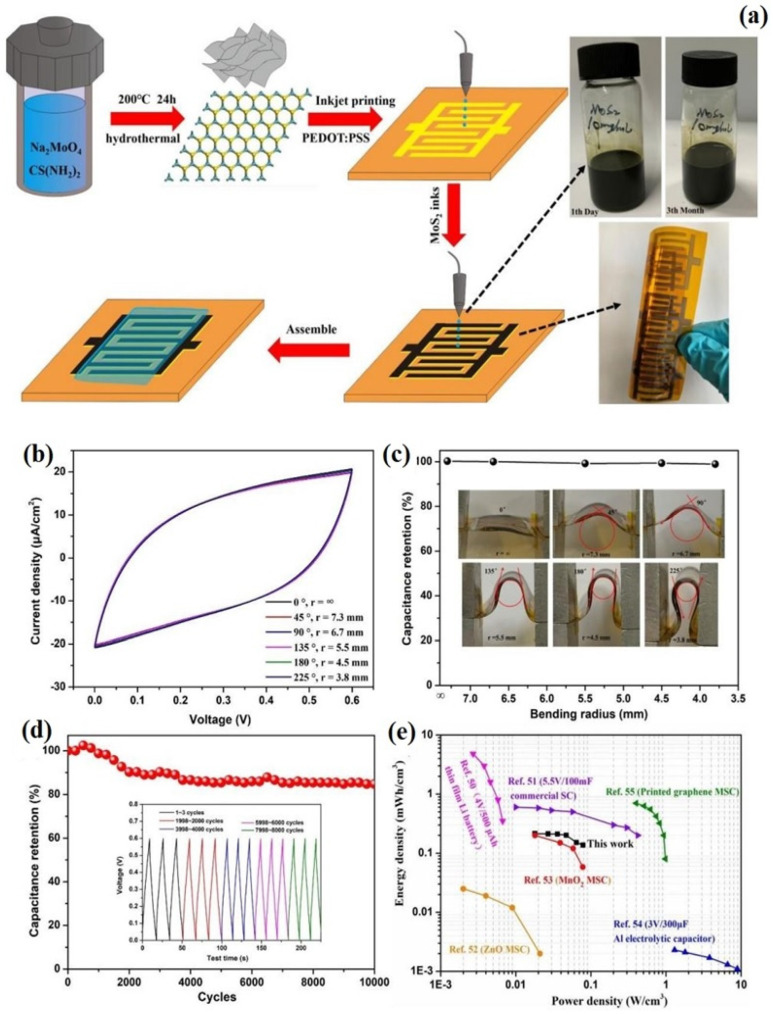
(**a**) Schematic representation of fabrication of MSC using MoS_2_ nanosheets, (**b**) Response of CV curves at a scan rate of 100 mV s^−1^ under various bending angles of MSC, (**c**) Demonstrating the flexibility of MSC under various bending radius, (**d**) Demonstrating the cyclic stability of MSC at 10 μA cm^−2^ up to 8000 cycles, (**e**) Comparing the performance of fabricated flexible MSC with the other devices using Ragone plot. Adopted from [207]. Copyright 2020 ACS Applied Materials & Interfaces.

Manuraj et al. developed a symmetric supercapacitor utilizing MoS_2_ as an active material with an impressively large mass loading. The process of growing MoS_2_ nanowires was conducted over Ni foam through a basic hydrothermal technique. The MoS_2_ nanowire electrode prepared at 36 h with a bulk loading of 30 mg cm^−2^ showed a specific capacity of 244 F g^−1^ and displayed an energy density of 12.2 Wh kg^−1^ at 1 A g^−1^ in the symmetric configuration. Even after undergoing 9000 charge/discharge cycles at 5 A g^−1^, the capacitor managed to maintain almost 92% of its maximum capacitance [204]. A recent study reports the effective fabrication of an MoS_2_/Gr heterostructure using a cost-effective and simple strategy. The versatile and asymmetrical supercapacitor gadget demonstrated remarkable electrochemical performance, with a specific capacity of 208 F g^−1^ at 0.5 A g^−1^. It also has an impressive specific energy of 65 Wh kg^−1^ and a power density of 0.33 kW kg^−1^. Moreover, the device maintained 86.5% of its electrochemical capacitance in spite of undergoing 10,000 cycles, demonstrating its exceptional long-term durability [205]. The article describes the preparation and characterization of a high-performance flexible and self-sustaining aerogel film electrode made of MoS_2_/SWCNT/CNF that is highly efficient. The electrode was employed in the creation of an all-solid-state supercapacitor that is both flexible and symmetric. The specific capacity and specific energy of the supercapacitor electrode were observed to increase as the amount of MoS_2_–SWCNT was raised. It was observed that the substance containing the highest amount of MoS_2_-SWCNT in MoSCF_3_ was capable of achieving a specific capacity of 605.32 mF cm^−2^ at a speed of 2 mV s^−1^ and exhibited a capacitance of 30.34 F g^−1^ when the speed was reduced to 0.01 A g^−1^. At an area-specific power of 0.03 mW cm^−2^, MoSCF_3_ had a recorded area-specific energy of 35.61 mWh cm^−2^. After undergoing 10,000 cycles of both charging and discharging, the capacity retention rate for MoSCF_3_ was observed to be at 91.01% [206]. The creation of ultrathin electrodes using MoS_2_ nanosheets through inkjet printing marks a significant advancement in the production of in-plane microsupercapacitors that are flexible and solid-state (Figure 9). These capacitors possess a high capacitance, with a maximum energy density of 0.215 mWh cm^−3^ and a high-power energy density of 0.079 W cm^−3^, making them suitable for various applications. In addition, they display strong cycle stability, retaining 85.6% of their capacitance even after undergoing 10,000 charge/discharge cycles. Another crucial advantage of these MSCs is that they are easy to scale up. This scalability makes it possible to fabricate large-scale, integrated circuits that can be used in a range of applications [207].

Chen et al. utilized a laser etching technology to directly etch the self-supporting MXene-MoS_2_ film for micro-supercapacitor (MSC) production. The micro-supercapacitor exhibited a maximum specific capacitance of 173.6 F cm^−3^ at a rate of 1 mV s^−1^. Moreover, it demonstrated a high energy density of 15.5 mWh cm^−3^ and a high power density of 0.97 W cm^−3^. After undergoing 6000 cycles of charge/discharge and being bent up to 150 °C, the specific capacitances of the MSC remained at approximately 98% of its initial value, highlighting the robustness and longevity of the MSC device [208]. The flexible conducting film with mesh-like silver networks was coated using a spin coating process with a dispersion of MoS_2_ that had been exfoliated electrochemically. Consequently, the composite electrode showed a significant areal capacity of 89.44 mF cm^−2^ at a current density of 6 mA cm^−2^. The electrode has an impressive energy density of 12.42 µWh cm^−2^ and power density of 6043 µW cm^−2^ at a current density of 6 mA cm^−2^ while used in a symmetric supercapacitor cell layout [209]. The future scope of MoS_2_ and its composites for flexible supercapacitors holds great potential in enhancing the performance, stability, and integration of energy storage devices. Continued research in this area will enable the development of more efficient and reliable energy storage systems. The electrochemical performances of several other MoS_2_ nanocomposite electrodes for FSC applications are listed in Table 2.

### 3.3. Energy Conversion Applications

MoS_2_ has been widely studied for its potential applications in energy conversion reactions, including photovoltaic cells, hydrogen production, and catalytic reactions. Studies have shown that MoS_2_ can be used as a highly efficient and stable photo-absorber in solar cells. MoS_2_ has shown excellent activity, stability, and durability as a catalyst in hydrogen evolution reactions. Apart from that, MoS_2_ has also been found to be effective in many other catalytic reactions, such as CO_2_ reduction, water splitting, and oxygen reduction reactions [219,220].

#### 3.3.1. Carbon Dioxide (CO_2_) Reduction

MoS_2_ is a promising material for CO_2_ reduction reactions, and ongoing research is focused on improving its conductivity and catalytic activity to make it comparable to other noble metal-based catalysts. With further advancements in this field, MoS_2_ could become an attractive alternative for sustainable CO_2_ conversion technologies [221]. Asadi et al. investigated that the edges of MoS_2_, specifically the inclined or sharp edges, are particularly effective at adsorbing CO_2_ and reducing it to CO. The study found that the inclined edges of MoS_2_ have a higher density of active sites for CO_2_ adsorption and reduction compared to the flat surfaces [222]. Recent research has shown that the concentration of sulfur vacancies in MoS_2_ materials can have a significant impact on their electrocatalytic performance for the reduction of CO_2_. Specifically, the presence of these vacancies can lead to lower overpotentials during the CO_2_ reduction reaction (CO_2_RR) and potentially offer a more efficient means of converting CO_2_ into valuable fuels and other chemicals. To better guide the design of these materials, researchers have used S K-edge x-ray absorption spectroscopy to identify the unique spectral signature associated with sulfur vacancies in MoS_2_. These findings offer promising avenues for the development of more efficient CO_2_RR electrocatalysts by leveraging the unique properties of sulfur-deficient MoS_2_ materials [223]. Linghu et al. investigated the catalytic potential of single-atom catalysts with low dimensionality for reducing CO_2_ to CH_4_. The research primarily examines how transition metal-modified 1T′-MoS2 monolayers promote stability and decrease CO_2_ through their catalytic mechanisms. The findings indicate that introducing transition metals in 1T′-MoS_2_ can expand the range of CO_2_RR products. Upon evaluating different catalysts, it was noted that Ru@1T′-MoS_2_ and Pt@1T′-MoS_2_ display the ability to stimulate the conversion of CO_2_ into CH_4_ and CH_3_OH, with relatively low limiting potentials of −0.56 and −0.73 V, respectively [224]. MoS_2_ has exposed edges that have a tendency to attract CO_2_ molecules and facilitate the catalytic conversion of CO_2_ to CO. This is achieved through the activation of the CO_2_ molecule by two Mo atoms located next to each other, causing the C–O double bond to undergo reconstruction during the adsorption process. The initial reaction between protons and electrons (H^+^ + e^−^) occurring at the edges of MoS_2_ follows a distinct pathway compared to that of a transition metal catalyst. It was further proved that the release of CO from the edges of MoS_2_ happens due to a distinct diffusion process for the CO atoms that have been adsorbed [225].

Recent research investigations have demonstrated that aqueous electrolytes containing single-crystal MoS_2_ or thin-film MoS_2_ electrodes can effectively reduce carbon dioxide, yielding 1-propanol as the primary CO_2_ reduction product. Furthermore, the predominant reduction process occurring at these electrodes is the reduction of water into hydrogen gas [226]. Khan et al. demonstrated that the addition of MoS_2_ layers onto NiTiO_3_ nanofibers could enhance the CO_2_ conversion yield significantly. In the study, the optimized combination yielded CO and CH_4_ at a rate of 130 and 55 μmol g^−1^ h^−1^, respectively, which is remarkably higher than those achieved from pure NiTiO_3_ nanofibers. Specifically, the yields were 3.8 times more for CO and 3.6 times more for CH_4_ compared to the pure nanofibers. This finding suggests that the tailored integration of the MoS_2_ layer on NiTiO_3_ nanofibers can potentially lead to the design of more efficient and effective CO_2_ conversion catalysts [227].

#### 3.3.2. Solar Cells

**Figure 10:**
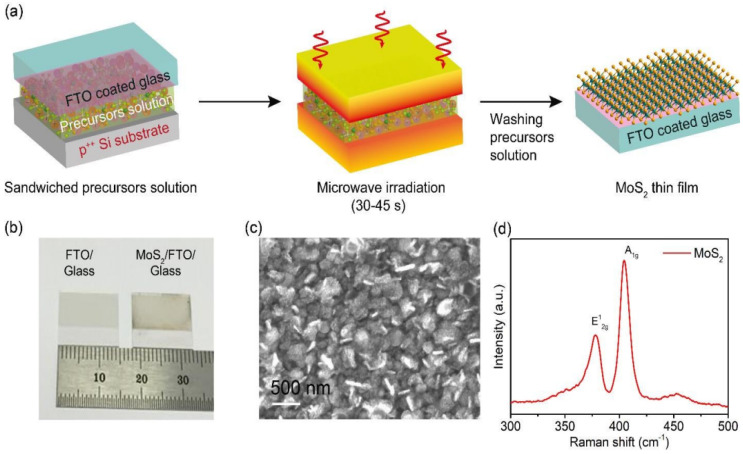
(**a**) Schematic representation of the synthesis of MoS_2_ thin film over the glass/FTO substrate using the microwave-assisted method, (**b**) MoS_2_ thin film created on a glass/FTO substrate and a bare glass/FTO substrate can be shown in a camera image, (**c**) image of a synthesized thin MoS_2_ film captured using High-resolution FESEM, (**d**) Raman peaks for E^1^ _2g_ and A_1g_ vibrational modes of MoS_2_ are displayed in the Raman spectrum of a thin film of MoS_2_. Adopted from [231]. Copyright 2019 Journal of Materials Chemistry A.

MoS_2_ has distinct chemical and structural properties that make it a highly desirable material for utilization in solar cells. Its outstanding carrier mobility facilitates the easy transfer of electrons and holes, leading to improved power conversion ability when employed in solar cells [228]. Tsai et al. successfully created a high-efficiency photovoltaic device by forming a type-II heterojunction with p-Si and MoS_2_ monolayers. The remarkable power conversion efficiency of 5.23% was achieved in a heterojunction photovoltaic device by utilizing a built-in electric field introduced near the interface, which aided in the separation of photogenerated carriers. This achievement represents a significant breakthrough in the use of monolayer TMDs for solar cell applications [229]. The integration of MoS_2_ into the ITO/p-Si/Ag solar cell structure has resulted in significant improvements in key performance factors, such as short-circuit current density, open-circuit voltage, and fill factor. These improvements have resulted in a remarkable increase in the conversion rate of the solar cell, from 1.1% to an impressive 4.6% [230]. The device with a structure of glass/FTO/MoS_2_/perovskite-po-spiro-OMeTAD/Au was utilized to produce perovskite solar cells (PSCs). These cells (Figure 10) demonstrated a power conversion rate of 13.1%, which is quite similar to the PSCs based on solid TiO_2_ and SnO_2_ electron transport layers [231]. Iqbal et al. described the effective activation of surface plasmon polaritons (SPPs) by fine-tuning the geometrical properties of MoS_2_-Au. The main objective is to improve the efficiency of photovoltaic cells that rely on plasmonics. This can be achieved by adjusting the slit widths (range encompasses wavelengths between 270 nm and 480 nm) and material thickness (ranging from 40 nm to 50 nm) while maintaining a constant periodicity of 720 nm for the unit cell [232].

Sattari et al. investigated the impact of a monolayer MoS_2_ film on the absorbance of CH_3_NH_3_PbI_3_, a favorable material for perovskite solar cells. The results show that the existence of the MoS_2_ film induces a rise in the absorbance of longer wavelengths [233]. Aymukhanov et al. described the findings from an investigation into how the presence of MoS_2_ nanoparticles affects electron transportation in the zinc oxide transport layer of a polymer solar cell. The researchers used impedance spectra measurements to analyze an organic solar cell and discovered that at a specific concentration, MoS_2_ nanoparticles enhance the lifetime of charge carriers and the diffusion coefficient in the composite film of zinc oxide [234]. The use of MoS_2_-assisted substrates for preparing perovskite films leads to improved crystallinity, which can be witnessed by enhanced photoluminescence and a prolonged emission lifetime. MoS_2_ quantum dots have a wider bandgap of 2.68 eV, which not only helps in hole collection but also prevents photogenerated electrons from moving to the hole transport layer [235]. The use of MoS_2_ nanoflakes as a hole transport interlayer can significantly enhance the stability and efficiency of Cs_2_AgBiBr_6_-based perovskite solar cells [236]. The hydrothermal technique was employed to deposit a blend of 1T and 2H phases of MoS_2_ onto an FTO substrate, resulting in the formation of thin films. The bandgap of MoS_2_ increased from 1.82 eV to 1.97 eV due to the change in shape, resulting in the superiority of mixed-phase MoS_2_ nanobelts. This makes them potential candidates for counter electrodes in platinum-free dye-sensitized solar cells at a lower cost [237].

#### 3.3.3. Hydrogen Evolution Reactions (HER)

MoS_2_ is a highly feasible substitute for valuable platinum in the catalysis of the hydrogen evolution reaction (HER). This is because it is abundant in nature, inexpensive, and has tunable electronic properties, as well as exceptional chemical stability. The potential to control the electronic properties of MoS_2_ during the catalysis process of HER could lead to fresh possibilities in the catalytic use of other transitional metal dichalcogenide materials and more [238]. The inclusion of heteroatoms is a useful approach for improving the catalytic behaviors of MoS_2_ through the impacts of interfaces and anions. This method can raise the number of active sites that facilitate speedy electron transfer. These advancements include the engineering of interfaces and anions, specifically for MoS_2_-Mo_2_C, MoS_2_-MoO_2_, MoS_2_-MoSe_2_, MoS_2_-MoP, and MoS_2_-MoN_x_. Enhanced conductivity and catalytic activity can be achieved through the introduction of heteroatom doping and the formation of heterostructures, leading to a substantial improvement in the performance of hydrogen evolution reaction [239]. An inkjet printing technique was used to create a Cu-supported 3D patterned catalyst consisting of MoS_2_/VP/RGO with partial 1 T-MoS_2_ and spatial configuration. This catalyst demonstrated remarkable catalytic performance, boasting an extremely minimal overpotential (51 mV at 10 mA cm^−2^, 126 mV at 100 mA cm^−2^), a very low Tafel slope (32 mV dec^−1^), and an exceptionally high cathodic current density [240]. The use of Er-MOF/MoS_2_ composite materials has proved to be a significant breakthrough in the field of electrocatalysis. These materials possess a porous structure, exposing more active sites and enhancing conductivity. This unique feature has resulted in exceptional hydrogen evolution reaction activity in acidic media, achieving a current density of 10 mA cm^−2^ with only 234 mV overpotential. Moreover, the Tafel slope of 54.3 mV dec^−1^ highlights the superior proficiency of these materials towards HER [241]. It has been found that reducing the growth time and temperature to sufficiently low levels enhances the performance of the HER catalysts. This promising observation offers a potential pathway for designing high-performance HER catalysts using MoS_2_-based compounds. This finding has significant effects on the progress of renewable energy technologies that rely on efficient HER [242]. The unique structure of the dendrite edge nanostructures in the monolayer MoS_2_ offers notable advantages over traditionally synthesized thermodynamic equilibrium MoS_2_. Due to the higher catalytic site densities, the dendrite MoS_2_ monolayer provides a highly efficient reaction surface area for HER. The Tafel slope of the dendrite MoS_2_ monolayer was significantly lower (59 mV dec^−1^) compared to the triangular MoS_2_ sample (97 mV dec^−1^), indicating that the dendrite MoS_2_ monolayer is more effective in facilitating HER [243].

**Figure 11:**
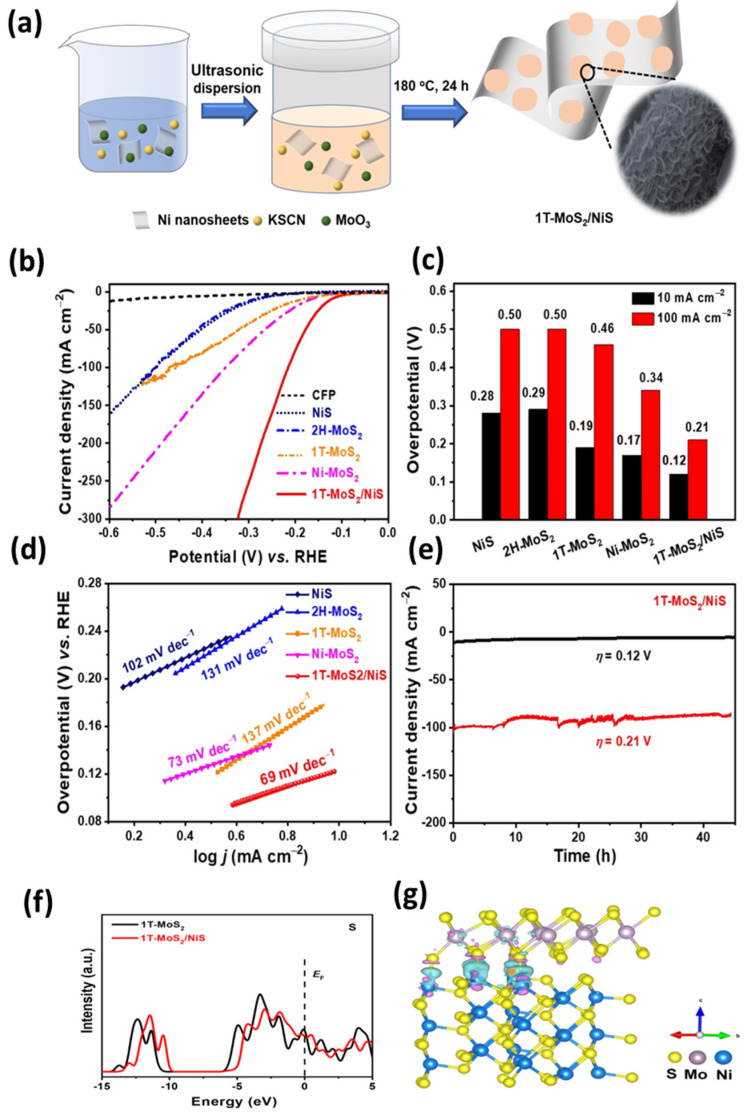
(**a**) Schematic representation of synthesis process of 1T-MoS_2_/NiS composite, (**b**–**e**) Electrochemical characterization of NiS, 2H-MoS_2_, 1T-MoS_2_, Ni-MoS_2_, and 1T-MoS_2_/NiS catalyst at 1.0 M KOH, (**b**) Polarization curves (without iR correction) (**c**) Overpotential at a current density of 10 mA cm^−2^ and 100 mA cm^−2^, (**d**) Tafel plots, (**e**) Durability tests of the 1T-MoS_2_/NiS catalyst at a constant potential of −0.12 V and −0.21 V vs. RHE, (**f**) Projected density of states of 1T-MoS_2_ and 1T-MoS_2_/NiS catalysts with the Fermi level set to zero, (**g**) The electron transfer process from NiS to 1T-MoS_2_ is represented through the side view of the charge density distribution of 1T-MoS_2_/NiS with cyan and pink colors indicate an increase and decrease in electron density, respectively. Adopted from [249]. Copyright 2022 Catalysts.

The CoCH nanosheets were utilized to create a three-dimensional self-supported cross-linked Co-MoS_2_ nanostructured HER catalyst. This catalyst has numerous active centers and the ability to transfer electrons quickly. Furthermore, the Co component is effective in activating the basal-plane sulfur atom in MoS_2_, making it the reactive center for HER [244]. The remarkable durability of the MoS_2_@Gr catalyst has been proven through extended experimentation in both acidic and alkaline electrolytes [245]. The research on morphology and spectroscopy suggests the production of excellent MoS_2_ nanosheets with metal ions that have been randomly doped. It is noteworthy to point out that Ni-MoS_2_ exhibited a superior HER performance in comparison to Co-MoS_2_ and Fe-MoS_2_ when considering other transition metals doping on MoS_2_. To achieve a current density of 10 mA cm^−2^, an overpotential of only −0.302 V vs. reversible hydrogen electrode (RHE) was needed for Ni-MoS_2_ [246]. The enhanced efficiency of HER observed in the MoS_2_ electrocatalyst doped with dual atoms is a result of alterations made to the electronic structure of the material. The dual atoms act as electron donors, and they effectively regulate charge on the S site, increasing the adsorption of H^+^. The almost negligible binding energy of H adsorption was discovered in the TM-Co co-doped layer of MoS_2_. It is highly noteworthy because it suggests that this substance could serve as a proficient electrocatalyst for HER and potentially replace platinum [247]. The Co, P-codoped MoS_2_, which is an optimized form of molybdenum disulfide, has shown remarkable catalytic activity. It exhibits a lower overpotential of 230 mV at a current density of 10 mA cm^−2^. The Tafel slope is smaller than 53 mV dec^−1^, which implies that the rate of HER is much faster compared to the pristine MoS_2_. Furthermore, the Co, P-MoS_2_ has also demonstrated excellent stability in acidic media, which is a crucial factor for the long-term use of catalysts in practical uses [248]. The 1T MoS_2_/NiS heterostructure exhibits higher efficiency in HER activity in comparison to 1T MoS_2_, particularly in 1.0 M KOH. At a current density of 10 mA cm^−2^, it attains a 0.12 V overpotential and a Tafel slope of 69 mV dec^−1^. This improvement is credited to interface engineering, which regulates the electronic states of the S sites in 1T MoS_2_, thus contributing to the boosted HER activity of the 1T MoS_2_/NiS heterostructure [249]. Wei et al. utilized interface engineering to create a heterostructure of 1T-MoS_2_/NiS, as shown in Figure 11. The resulting heterostructure demonstrated greater activity in terms of HER compared to that of 1T-MoS_2_, specifically in a 1.0 M KOH solution. At a current density of 10 mA cm^−2^, it achieved an overpotential of 0.12 V and had a Tafel slope of 69 mV dec^−1^. According to calculations based on density functional theory (DFT), the heterostructure of 1T-MoS_2_/NiS induced by interface engineering shows regulated electronic states of S sites in 1T-MoS_2_, which leads to an improved HER activity. This study suggests that adjusting the electronic structure through interface engineering could be an effective approach for enhancing the intrinsic activity of electrocatalysts [249].

#### 3.3.4. Oxygen Evolution Reactions (OER)

**Figure 12:**
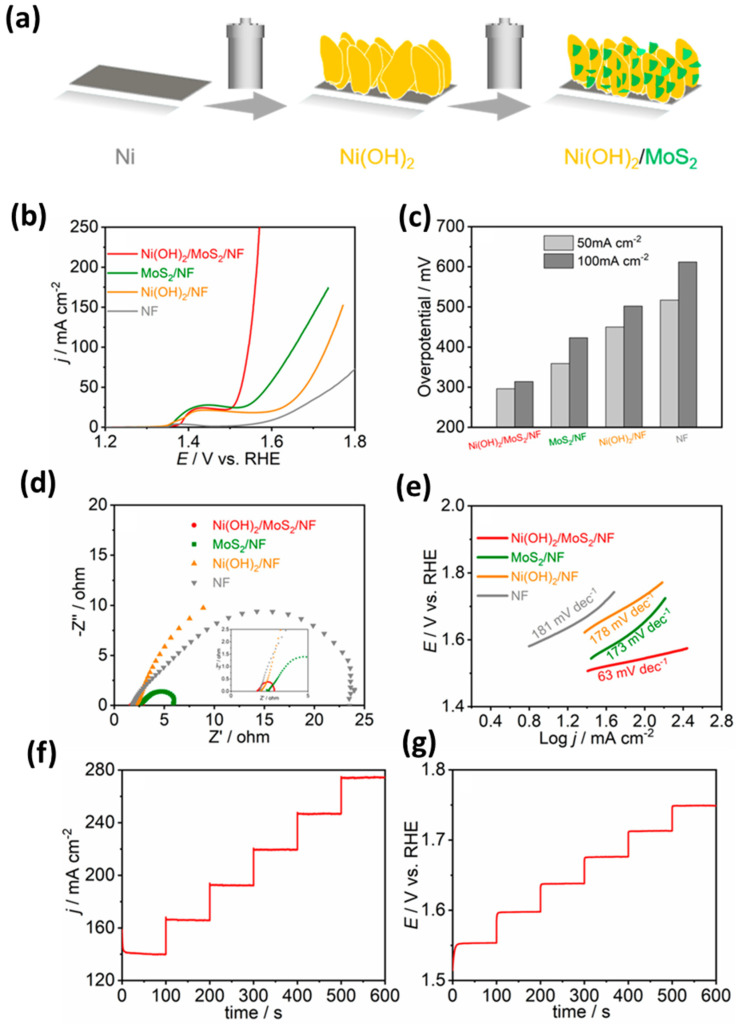
(**a**) Schematic representation of synthesis process of Ni(OH)_2_/MoS_2_/NF composite electrode, (**b**–**e**) Electrochemical characterization of Ni(OH)_2_/MoS_2_/NF, MoS_2_/NF, Ni(OH)_2_/NF and bare NF electrodes, (**b**) The polarization curves with iR corrected, (**c**) The overpotentials at 50 mA cm^−2^ and 100 mA cm^−2^, (**d**) Electrochemical impedance spectroscopy, (**e**) Tafel plots, (**f**) The multi-potential steps of Ni(OH)_2_/MoS_2_/NF electrode, (**g**) The multi-current steps of Ni(OH)_2_/MoS_2_/NF electrode. Adopted from [256]. Copyright 2022 Catalysts.

The oxygen evolution reaction (OER) is the electrochemical process of generating oxygen gas from water in the presence of an electric potential. It is an important reaction in various applications, including in the production of hydrogen fuel from water and in renewable energy generation systems such as electrolyzers and fuel cells. German, E. et al. reported that the poor performance of monolayer MoS_2_ in the oxygen evolution reaction could be attributed to the fact that the reaction intermediates HO* and HOO* bind less strongly compared to the intermediate O*, resulting in a higher overpotential of 1.13 eV. The overpotential on the Mo-edge is nearly the same as that on the pure MoS_2_ monolayer, while on the S-edge, the overpotential is significantly higher. This means the S-edge configuration is even less desirable for the OER [250]. Bao et al. created a heterostructure catalyst for OER on a Ti mesh substrate by merging MoS_2_ nanosheets with NiCo_2_O_4_ hollow spheres. The electrocatalyst has demonstrated outstanding OER performance in an alkaline setting, producing overpotentials of 313 and 380 mV and reaching 10 and 100 mA cm^−2^ [251]. The remarkable electrochemical characteristics of the hybrid nanomaterial, comprising 1T MoS_2_/Co_3_S_4_/Ni_3_S_2_, demonstrate its potential as a favorable option for energy conversion uses. The nanoarray is able to achieve low overpotentials of only 50 and 240 mV at a current density of 10 mA cm^−2^ for HER and OER, respectively. Furthermore, the nanoarray displays remarkable stability, making it a reliable and long-lasting option for applications in various electrochemical devices [252]. The SnO_2_@MoS_2_ heterostructures and the synergistic effects of MoS_2_ and SnO_2_ contributed to the improved catalytic activity in both HER and OER. The bi-functional electrode, which possessed these distinct characteristics, demonstrated remarkable performance in alkaline water electrolysis. It accomplished 10 mA cm^−2^ at a cell voltage of 1.57 V and retained 98.3% of its initial current after 300 h of continuous testing, exhibiting exceptional long-term electrochemical stability [253]. The unique 3D structure of MoS_2_ nanosheets anchored on Ni_9_S_8_ nanorods has shown promising results in the field of water splitting. As a result, the MoS_2_/Ni_9_S_8_ composite exhibits a low overpotential at 50 mA cm^−2^ for OER and 10 mA cm^−2^ for HER during the water-splitting process [254]. The electrocatalytic activity of cobalt-aluminum-layered double hydroxide can experience substantial improvement through the dual doping of MoS_2_ and Ce. As a result, CoAl LDH@MoS_2_ with 5% Ce exhibits a potential of 1.508 V at a current density of 10 mA cm^−2^, which is notably lower than the 1.733 V observed in CoAl LDH [255]. A novel Ni(OH)_2_/MoS_2_/NF electrocatalyst was synthesized using two-step hydrothermal and solvothermal methods shown in Figure 12. Exceptional OER performance in 1.0 M KOH electrolyte was observed, with a lower overpotential of 296 mV at 50 mA cm^−2^, along with remarkable durability. A thorough examination demonstrates that the improved catalytic activity results from the cooperative influence of Ni(OH)_2_ and MoS_2_, which provides more active sites for the catalyst. This also offers a dependable approach to implementing heterogeneous interface engineering for energy catalysis [256].

The single-atom catalytic mechanism utilized in TMO_6_@MoS_2_ (TM = Fe, Mn and Co) species creates active sites and orbitals that are responsive to adsorption. This allows for increased adsorption of oxidative intermediates and ultimately improves the catalytic activity of the OER. When compared to benchmark IrO_2_, TMO_6_@MoS_2_ species demonstrate superior electrocatalytic activity for the OER [257]. The combination of CoFeO_x_(OH)_y_ and MoS_2_ results in greater efficiency in converting water into oxygen through the process of oxidation. This occurs because the electronic interaction between the two materials increases the amount of energy available to convert water into oxygen, leading to improved activity and stability of the electrode. The resultant CFOMS-CP electrode offers a promising solution for efficient and reliable electrochemical OER applications [258]. The formation of heterojunctions has been demonstrated to be an effective method for enhancing the electrocatalytic activity of materials, particularly in the context of OER. In the case of MoS_2_, the incorporation of a graphene-like material, g-C_3_N_4_, has demonstrated significant improvements in OER performance due to the modification of the electronic structure. The high catalytic activity of the MoS_2_/g-C_3_N_4_ heterojunction can be attributed to the heteroepitaxial growth of the two materials, creating an interface between them. This electronic coupling enables efficient electron transfer between the materials, which promotes the OER process [259]. Elrahim et al. explored the fabrication of Co_3_O_4_–MoS_2_/Ni foam electrodes utilizing a vacuum kinetic spray method with microparticles of MoS_2_ and Co_3_O_4_. The research findings suggest that the addition of MoS_2_ in a gradual manner increases the OER activity, which is attributed to improved charge transfer kinetics [260]. The use of AlCo_3_-MoS_2_ substrate in electrocatalytic OER applications has shown to be highly effective due to the production of M−O (M=Al, Co and Mo) species with oxygen vacancies. These species play a crucial role in initiating the surface self-reconstruction of pre-catalysts, resulting in the formation of new and highly active facets. The oxygen vacancies created on the substrate also assist in improving the electronic conductivity of the catalyst, further enhancing its electrocatalytic performance [261].

Mixed oxide-based lithium electrode batteries are widely favored for their stability and capacity retention after numerous cycles. In contrast, MoS_2_-based LIBs have impressive specific capacities but require further improvement in cycling stability and cost-effectiveness to become more competitive. The rising cost and high demand for Li-ion batteries have sparked interest in exploring Na-ion-based batteries as a viable alternative. In order to meet commercial needs, it is important to conduct more thorough research on electrode materials, electrolytes and current collectors for SIBs. This can accelerate the path toward commercialization by enhancing our understanding of storage capacity and the preparation of electrode materials. MoS_2_ has become increasingly popular as a material for supercapacitors due to its decent charge storage capacity. However, it still falls behind state-of-the-art materials like graphene or mixed oxide, which possess considerably higher specific surface areas for charge storage. Research is being conducted on composite materials that contain MoS_2_ to enhance their storage capacity. However, the manufacturing cost of these composites is high, and there is a loss in electrochemical performance that needs to be resolved. There are still challenges to be addressed when it comes to integrating MoS_2_ into solar cells, such as ensuring its stability and scalability, but the potential benefits are great. With continued research and development, MoS_2_-based solar cells could become a major player in the renewable energy market. The ongoing research on HER catalysts is primarily centered on substituting costly noble metal catalysts with more economical and easily accessible materials. The ultimate objective for HER catalysts materials is to reduce the Tafel slope and enhance the current density. Recently, there has been a shift towards utilizing MoS_2_-based electrocatalysts as a means to enhance catalytic performance and eliminate the use of noble metals. These catalysts have demonstrated comparable long-term stability in HER acidic solutions as platinum-based electrocatalysts. However, the existing MoS_2_-based catalysts have limited ability to produce high current densities, leading to impractical Tafel slopes. On the contrary, electrodes based on MoS_2_ are not yet as efficient and stable over repeated cycles compared to the current leading technology. Despite its high electrical conductivity, 1T-MoS_2_ is challenging to prepare as it involves the use of hazardous and air-sensitive materials. Advanced equipment like glove boxes and autoclaves are necessary, which makes its manufacturing expensive and difficult to scale. Therefore, finding a safer method to prepare conductive MoS_2_ would be highly advantageous in overcoming the limitations of electrical conductivity.

## 4. Challenges and Future Directions

Despite the promising properties of MoS_2_, there are certain challenges that limit its commercial applications. Synthesizing MoS_2_ on a large scale in a cost-effective way is still a major challenge. The conductivity of 2H MoS_2_ and the stability of 1T MoS_2_ has to be addressed in the future. The outstanding properties of 1T MoS_2_ were more suitable for commercial energy storage applications in hybrid supercapacitors. More research has been focused on increasing the production of 1T MoS_2_ using several synthesis techniques and stability [38,109]. The properties of MoS_2_ also depend on its quality and size, which can be influenced by the synthesis method, additives, and processing conditions. To achieve high-quality MoS_2_, its synthesis method needs careful consideration, as even minor variations can impact its properties. The preferred method for producing an MoS_2_ layer that is uniform, of high quality, and covers a large surface area is chemical vapor deposition (CVD). However, the CVD process has certain limitations, such as high-temperature requirements and limited control over thickness and size. Besides the synthesis method, additional processing steps are also required to optimize MoS_2_’s properties. These additional steps include surface modification, doping, and dispersion in various solvents. Surface modification can improve MoS_2_’s properties by increasing its stability, biocompatibility, and electrical conductivity. Doping of MoS_2_ with other elements can enhance its electronic and optical properties, as well as improve its catalytic activities. Dispersion of MoS_2_ in solvents can increase its uniformity, stability, and availability for further processing. Apart from the crystal quality and size, controlling the particle size of MoS_2_ is also crucial for numerous applications, including energy storage and catalysis. The particle size of MoS_2_ affects its surface area and hence its reaction kinetics, as well as its interaction with other materials. Therefore, several techniques have been developed to control MoS_2_ particle size, including hydrothermal synthesis, microwave-assisted synthesis, and electrode deposition. In conclusion, the particle size, crystalline structure, and purity of MoS_2_ are critical factors that need careful consideration to harness its full potential in various applications. A better understanding of the synthesis and processing methods can maximize MoS2’s properties, ultimately leading to its widespread usage in various fields ranging from energy to biomedicine [262].

To overcome the stability issue, researchers are making enormous efforts to enhance the metallic phase of MoS_2_. There have been significant advancements in this area, including the usage of transition metal doping and chemical treatment methods to improve the cyclic stability of MoS_2_ in the metallic phase. Despite these advancements, there is still a need for further research to improve the stability of MoS_2_ in its metallic phase. The study of MoS_2_’s stability has both scientific and practical applications, and addressing these issues will open up new avenues for its use in various fields, including electronics, energy storage and catalysis [263]. MoS_2_ has good electrochemical properties in energy applications, but it seems to be low as compared to Nobel metals [264]. The study of pristine MoS_2_ was almost completed, but the study of its nanocomposites with noble metals, carbon and metal oxides/sulfides with outstanding characteristics in energy storage/conversion applications needs further research [39,265]. The restacking of the layers in MoS_2_ reduces its effectiveness in certain applications. Additionally, MoS_2_ undergoes significant volume expansion during electrochemical intercalation, which can lead to structural instability or even failure. Another issue is its poor intrinsic electronic conductivity, which hinders its ability to efficiently conduct electrical charges. While researchers are actively exploring ways to mitigate these challenges, further developments are needed to fully unlock the potential of MoS_2_ for practical and commercial use [266,267].

The several research gaps in the understanding and development of MoS_2_ and its composites are summarized.

1. Synthesis methods: there is a need for developing simple, cost-effective synthesis methods for large-scale production of MoS_2_ and its composites, especially those with controlled morphology and structure;
2. Stability and durability: it is crucial to investigate the stability and durability of MoS_2_ and its composites under various environmental and mechanical conditions, which would provide insights into the potential applications of these materials;
3. Composites design: developing novel and effective composite designs by incorporating MoS_2_ with other materials can lead to enhanced properties, such as mechanical strength, electrical conductivity, and wear resistance. However, there is still a need to identify the optimum composition and processing conditions for achieving the desired properties;
4. Characterization: there are still gaps in the understanding of the structural and compositional properties of MoS_2_ and its composites, particularly for those with multiple layers and complex architectures;
5. Applications: the full potential of MoS_2_ and its composites in various applications, including energy storage, catalysis, electronics, and biomedicine, is yet to be explored. It is necessary to investigate the performance and reliability of these materials in real-world applications.

Overall, bridging these research gaps can significantly advance our understanding and utilization of MoS_2_ and its composites in various fields.

## 5. Conclusions

Our review highlights MoS_2_ as an emerging material for future energy applications. MoS_2_ stands out due to its economic cost, copious availability, and uncomplicated synthesis process. Moreover, its distinctive photocatalytic and electrocatalytic characteristics and structure have created opportunities for its utilization in energy generation reactions and energy storage devices. In addition, the characteristics of its structure, photocatalysis, and electrocatalysis have made it suitable for utilization in energy storage devices and energy generation applications. The chemical exfoliation approach is a convenient choice for industrial manufacturing procedures because it can be expanded effortlessly to produce MoS_2_ nanosheets on a large scale. The 1T MoS_2_ metallic phase has several desirable properties that make it suitable for use in various applications. As a result of its excellent conductivity and relatively weak van der Waal forces between layers, it is a suitable material for use as a Li-ion battery anode because Li-ions can easily intercalate between the layers. It also has a large area and superconductivity, making it perfect for use in supercapacitors. Its ability to restack easily is an added advantage. The high electrocatalytic and photocatalytic activity of MoS_2_ makes it useful in solar cells and hydrogen/oxygen evolution reactions. It has the potential to minimize CO_2_ and generate energy, making it a promising solution for environmentally-friendly energy production and mitigation of CO_2_ levels in our surroundings. The use of MoS_2_ in various technological applications is still being explored and researched, and it holds immense potential for the development of novel and advanced technologies. As the demand for sustainable and environmentally friendly solutions grows, the versatility and unique properties of MoS_2_ make it a valuable material in the advancement of various technological fields. It is evident that the potential of MoS_2_ is limitless and future research in this area is necessary to explore its full potential and benefit society as a whole.

## Figures and Tables

**Table 1:** Comparison of various synthesis methods of MoS_2_.

Synthesis Method	Merits	Demerits
Mechanical exfoliation	High-quality crystal structure; Same crystal structure as their bulk; Simple method; No chemical reaction involved.	Less yield produced; Substrate assistance is needed; Thickness of the layer was not controllable.
Ball milling	High yield/Massive production; High surface area; Reaction parameters are controllable.	The inert condition required; Yield has a few-layered thickness.
Liquid phase exfoliation	High yield/Massive production; Scalable method; Low-cost method.	Toxic organic solvents involved; Yield has a few-layered thickness.
Chemical vapor deposition	High-quality crystal with purity; Size and thickness are scalable; High surface area films; High deposition rate.	Solid substrate assistance is needed with a vacuum; High operating temperature; Possibility of toxicity of precursors.
Physical vapor deposition	Atomic-level control of chemical deposition; Safer than CVD.	Low deposition rate; Requirement of annealing time; Line-of-sight deposition.
Wet chemical synthesis	High yield with low cost; Easy hybridization with other nanomaterials; Controllable size and shape.	Large degree of agglomeration may occur, Difficult to obtain nanoparticles with a single layer.

**Table 2:** MoS_2_ nanocomposites as electrode materials for FSC applications.

Electrodes	Synthesis Method	Specific Capacitance	Retention after Bending/ Twisting	Retention Rate (Cycles)	Energy Density	Power Density	Ref.
MoS_2_/Gr	Hydrothermal method	208 F g^−1^	NA	86.5% (10,000)	65 Wh kg^−1^	0.33 kW kg^−1^	[205]
MoS_2_/NGQDs/ HCNT@CC	Hydrothermal method	1893 mF cm^−2^	NA	86% (2500)	673 μWh cm^−2^	5687 μW cm^−2^	[210]
CC-CNC@ MoS_2_	Hydrothermal method	120.7 mF cm^−2^	NA	88.2% (10,000)	0.016 mWh cm^−2^	8.3 mW cm^−2^	[211]
MoS_2_/NCC	Plasma method	3834.28 mF cm^−2^	NA	83.3% (10,000)	138.12 µWh cm^−2^	7417.33 µW cm^−2^	[212]
1T-MoS_2_/ Cu(OH)_2_@CFP	wet-mold papermaking technology	1124 mF cm^−2^	NA	90.8% (20,000)	0.130 mWh cm^−2^	0.375 mW cm^−2^	[213]
MoS_2_@SSM	DC sputtering method	214.90 F g^−1^	92%	88% (3000)	28.05 Wh kg^−1^	0.26 kW kg^−1^	[214]
MoS_2_-AlN@SS	binder-free sputtering method	372.35 F g^−1^	95%	93% (5000)	28.05 Wh kg^−1^	0.26 kW kg^−1^	[215]
MoS_2_@CoS_2_	calcination and sulfuration	950 F g^−1^	NA	94.6% (10,000)	33.94 Wh kg^−1^	1040 W kg^−1^	[216]
MoS_2_-Cu_3_N	Magnetron Sputtering	215.47 F g^−1^	91%	90% (2000)	30 Wh kg^−1^	138 W kg^−1^	[217]
MoS_2_/PANI/CNT	Sonication method	245 F cm^−3^	NA	80% (2000)	0.013 Wh cm^−3^	1.0 W cm^−3^	[218]

Note: NA—Information not available.

## Data Availability

Not applicable.
